# Diversity of innate immune cell subsets across spatial and temporal scales in an EAE mouse model

**DOI:** 10.1038/s41598-018-22872-y

**Published:** 2018-03-23

**Authors:** Céline Caravagna, Alexandre Jaouën, Sophie Desplat-Jégo, Keith K. Fenrich, Elise Bergot, Hervé Luche, Pierre Grenot, Geneviève Rougon, Marie Malissen, Franck Debarbieux

**Affiliations:** 1 Institut des Neurosciences de la Timone, Aix-Marseille Université and CNRS UMR7289, Marseille, France; 2 Centre Européen de Recherche en Imagerie Médicale, Aix-Marseille Université, Marseille, France; 3 Centre d’Immunophénomique CIPHE (Phenomin), Aix-Marseille Université UMS3367, INSERM, US012, CNRS UMS3367 Marseille, France; 4 Centre d’Immunologie de Marseille-Luminy, Aix Marseille Université UM2, INSERM, U1104, CNRS UMR7280 Marseille, France; 5 Aix-Marseille Université, NICN, CNRS, UMR7259 Marseille, France; 6 Service d’Immunologie, Pôle de Biologie– Hôpitaux de Marseille, Marseille, France; 7 Present Address: Faculty of Rehabilitation Medicine University of Alberta Edmonton, Alberta, T6G 2G4 Canada

## Abstract

In both multiple sclerosis and its model experimental autoimmune encephalomyelitis (EAE), the extent of resident microglia activation and infiltration of monocyte-derived cells to the CNS is positively correlated to tissue damage. To address the phenotype characterization of different cell subsets, their spatio-temporal distributions and contributions to disease development we induced EAE in Thy1-CFP//LysM-EGFP//CD11c-EYFP reporter mice. We combined high content flow cytometry, immunofluorescence and two-photon imaging in live mice and identified a stepwise program of inflammatory cells accumulation. First on day 10 after induction, EGFP^+^ neutrophils and monocytes invade the spinal cord parenchyma through the meninges rather than by extravasion. This event occurs just before axonal losses in the white matter. Once in the parenchyma, monocytes mature into EGFP^+^/EYFP^+^ monocyte-derived dendritic cells (moDCs) whose density is maximal on day 17 when the axonal degradation and clinical signs stabilize. Meanwhile, microglia is progressively activated in the grey matter and subsequently recruited to plaques to phagocyte axon debris. LysM-EGFP//CD11c-EYFP mice appear as a powerful tool to differentiate moDCs from macrophages and to study the dynamics of immune cell maturation and phenotypic evolution in EAE.

## Introduction

In Multiple Sclerosis (MS) immune cells attack leads to widespread demyelination, axon damage, and neurological deficits. Experimental autoimmune encephalomyelitis (EAE) is the most relevant and commonly used animal model to study MS^1–3^. In MS patients and EAE mice, monocytes accumulate in demyelinated areas and their numbers correlate to tissue damage^4^. However, the cellular mechanisms linking neuroinflammation and axonal degeneration remain elusive. To date, many studies have examined the role of adaptive immunity in both EAE^5,6^ and MS. Comparatively, the role of innate immune system is poorly understood although it might contribute to both initiation and progression of the disease.

Monocyte derived cells and resident microglia are indistinguishable on tissue sections based on morphological features or surface marker expression; all populations can carry out phagocytosis and chemokine secretion. These cell types were often discussed as a single functional macrophage population, but today microglia and macrophages are recognized as ontogenetically distinct: microglia derive from yolk-sac progenitors during embryogenesis and self-renew in the adult parenchyma^4,7,8^, whereas macrophages continuously differentiate throughout postnatal life from bone marrow hematopoietic precursors^9–11^. Only some macrophages present in the non-parenchymal area of the central nervous system (CNS) have been recently shown to be long lived cells of embryonic origin similar to microglia^12^.

These differences in developmental origin suggest that bone marrow-derived macrophages and microglia exert different functions and respond differently to the same environmental stimuli in pathological processes^13^. Using serial block-face scanning electron microscopy, ultrastructural features have been used to distinguish monocytes derived cell populations in the EAE model^14^. However, lack of information on spatio-temporal localization and phenotype of infiltrating monocytes stands as a limitation. Yet, examining inflammatory cell subpopulations in precise time windows might lead to a major therapeutic approach. It is therefore crucial to obtain a more detailed understanding of cellular events of innate immune response inside the CNS.

In this study, we phenotyped innate immune cells throughout the development of EAE and correlated these findings with imaging data on individual mice. To this end, we applied the recent improvements in marker selection and gating strategies to EAE-induced Thy1-CFP//LysM-EGFP//CD11c-EYFP reporter mice to better define myeloid lineages at steady state and during inflammation^15,16^. The immune, neuronal and vascular compartments were non-invasively, recurrently observed using two-photon imaging of spinal cord (SC)^17–19^. Whereas demyelination and axonal damages are important causes of the functional deficits observed in multiple sclerosis and EAE^3,20^, and having access to mice with Thy1-CFP fluorescent axons, we specifically studied the correlation between axonal damage and temporal and spatial recruitment of subsets of immune cells in relation to clinical scores. This *in vivo* imaging approach also allowed us to observe the morphological changes and motility of fluorescent immune cells. Altogether, this unique toolbox and dataset constitute a well-defined model allowing testing and deciphering the effect of therapies for MS at the cellular level.

## Results

### Gating strategy applied to evaluate qualitatively and quantitatively all immune cells contained in SC and brain during EAE

To document the nature of the immune response and the degree of heterogeneity found in the innate immune cell populations during EAE progression, we performed multi-parametric flow cytometry analyses on brain and SC tissues of MOG-induced EAE (MOG.CFA.PTX), CFA.PTX treated (CFA.PTX control) and PBS injected (PBS control) mice. The clinical scores of all mice were evaluated daily. At days 8, 13 and 17 after EAE induction we collected and dissociated independently the brain and SC of individual animals of each group after removing circulating blood by PBS perfusion. Cell suspensions were labeled using a mix of 15 fluorescent antibodies for extensive characterization of immune cells infiltrating the CNS with an emphasis on myeloid cells. This strategy was applied previously in a mouse glioblastoma model^21^ and is adapted from gating strategies developed for mouse skin^15^, muscle and lymph nodes^22^ or gut mucosa^23^.

**Figure 1:**
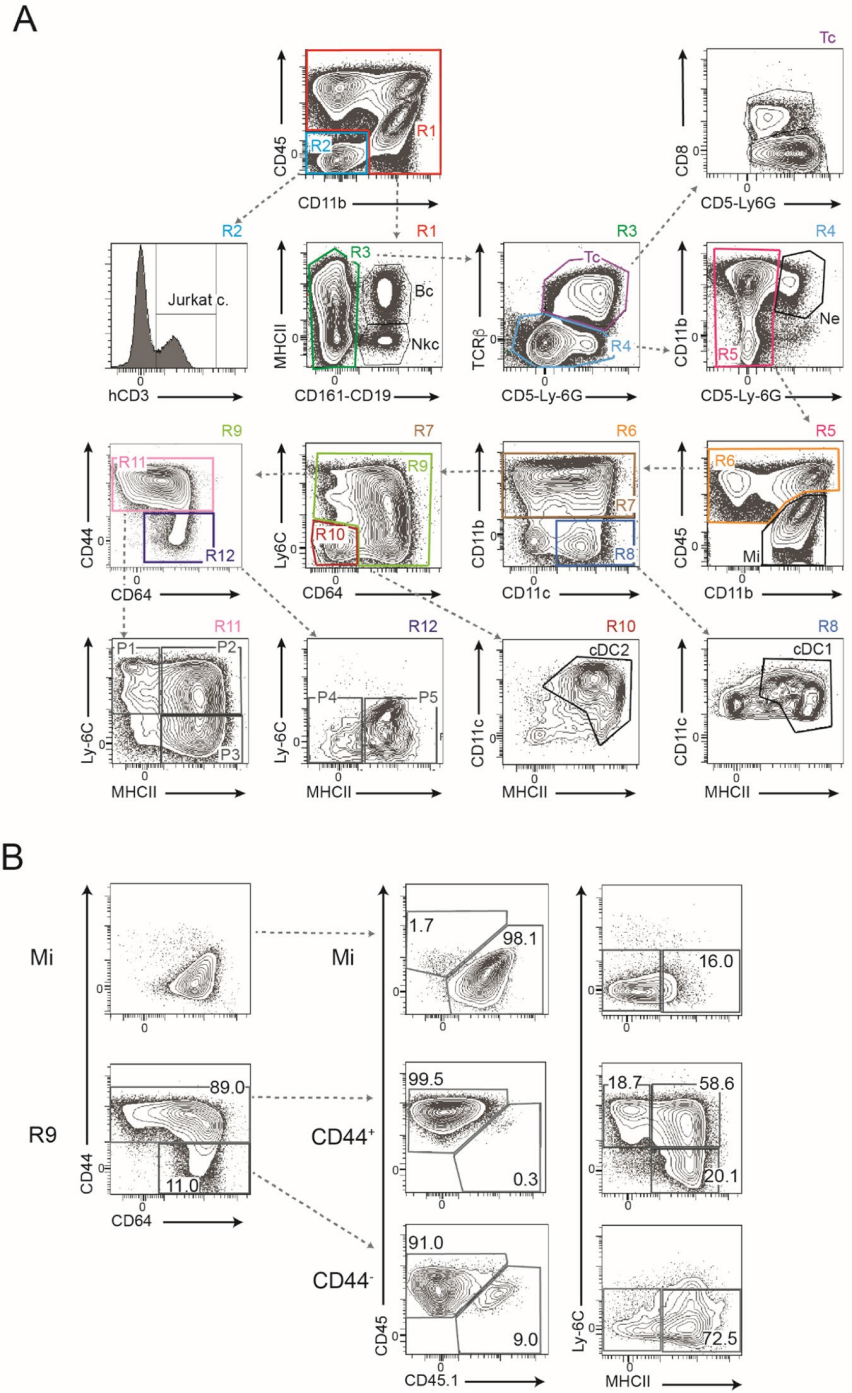
Gating strategy used for analyzing all immune cells within the CNS at steady state or during inflammatory conditions. (**A**) Starting from the upper left panel and going down following the arrows in dotted lines, we gated successively: CD45^+^ cells (R1) and human Jurkat cells (hCD3^+^)in R2, B cells (Bc) and NK cells (NKc) in R1, TCRαβ^+^ T cells in R3 (Tc, further subdivided in CD8^+^ and CD8^−^ Tc), neutrophils (Ne) in R4, microglia (Mi) in R5, monocytes P1 and moDCs P2-P3 in R11 and P4-P5 macrophages in R12. Conventional DC were gated in R10 (cDC2 or CD11b^+^DC) and R8 (CD11b^−^DC or cDC1). R1 to R12 in the contour plots indicate the number of the corresponding gate. For each contour plot the represented gate is indicated on the right side above the plot. (**B**) Validation of microglia and monocyte-derived cell gating using bone marrow chimeric mice. Top 3 panels represent microglia (Mi). The 5 lower panels represent monocyte-derived cells (R9). For each category, the original gating (CD44-CD64 plot) is shown on the left, the recipient (CD45.1) versus donor origin of the population in the middle and the maturation status Ly-6C-MHCII plot on the right. The percentage is indicated in each gate. (**A**) is representative of brain at day 17 and (**B**) of brain at day 15.

As depicted in Fig. 1A, among live, single Sytox Blue-negative cells, we first selected all CD45^+^ hematopoietic cells (R1). CD45^−^ cells (R2) contained the human CD3^+^ Jurkat cells introduced for calibration. The B cells (CD19^+^ MHCII^+^) and NK cells (CD161^+^ MHCII^−^) found in the CD45^+^ hematopoietic cells (R1 gate) were excluded. Among the remaining non-B, non-NK cells, αβ T cells were excluded from further analysis: they correspond to the R3 gate (TCRβ^+^. CD5^+^; Tc) and can be subdivided into CD8^+^ and CD8^−^ (CD4^+^ Tc) T cells. Neutrophils (CD11b^+^ Ly-6G^+^) were identified in the R4 gate. The remaining non-B, non-T, non-NK, non-neutrophils cells found in the R5 gate corresponded to microglial cells, macrophages, monocytes, monocyte-derived cells and dendritic cells. Relative levels of CD45 discriminated between resting (CD45^dim^) and activated (CD45^int^) microglial cells (Mi) from blood derived infiltrated cells (CD45^high^; R6 gate)^24,25^.

The R6 gate can be subdivided into two disctinct populations CD11b^+^ (R7) and CD11b^−^ CD11c^+^ (R8) cells. Among CD11b^+^ cells, a Ly6C-CD64 plot identified a large population (R9) consisting of monocytes, monocyte-derived DC (moDCs) and macrophages^15^.

Monocytes, moDCs and macrophages share many markers making their discrimination difficult in the CNS under EAE inflammation. It was however reported that CD44^+^ cells corresponded to monocytes and moDCs and CD44^−^ cells to macrophages^8,26^. We indeed validated CD44 in our panel using bone marrow chimera involving CD45.1 recipient mice and bone marrow transfer cells of CD45.2 origin. EAE was induced in the chimeric mice and the immune cells present in the brain were analyzed either at steady state (data not shown) or at the onset of disease (Fig. 1B). We restricted our analysis to (1) microglia, as defined above in R5 gate and (2) to monocytes, monocyte-derived cells and macrophages, all contained in R9 gate. Cells in R9 were split into CD44^+^ (R11) and CD44^−^ (R12) gates, while microglia cells were all CD44^−^. Microglia, R11 and R12 cells were then analyzed for their host or bone marrow origin. As shown in Fig. 1B, microglia cells were exclusively of CD45.1^+^ host origin and showed a Ly6C^−^MHCII^lo^ phenotype as previously described^12^. Similar to other studies^15,21^, CD44^+^ cells present in the R9 gate were exclusively from donor bone marrow origin and included monocytes (P1 cells: Ly6C^+^ MHCII^−^) and (moDCs) (P2 and P3 cells: Ly6C^+/−^ MHCII^+^). In contrast, CD44^−^ cells present in the R9 gate were comprised of Ly6C^−^ MHCII^−/hi^ macrophages (denoted as P4-P5 cells in)^15^, most of which (91%) were of bone marrow origin. Therefore, the analysis of chimeric mice validated the commonly used CD45^lo^ microglia gate and the CD44 marker to differentiate moDCs from macrophages in the CNS both under steady state and under strong inflammation. Finally, we identified conventional dendritic cells (cDC) as CD11c^+^ MHCII^high^ CD11b^+^ cDC2 (R10 gate) and as CD11c^+^ MHCII^high^ CD11b^−^ cDC1 (R8 gate), also known as XCR1^+^ DCs.

### Comprehensive analysis of innate immune cells in blood and SC during EAE progression

**Figure 2:**
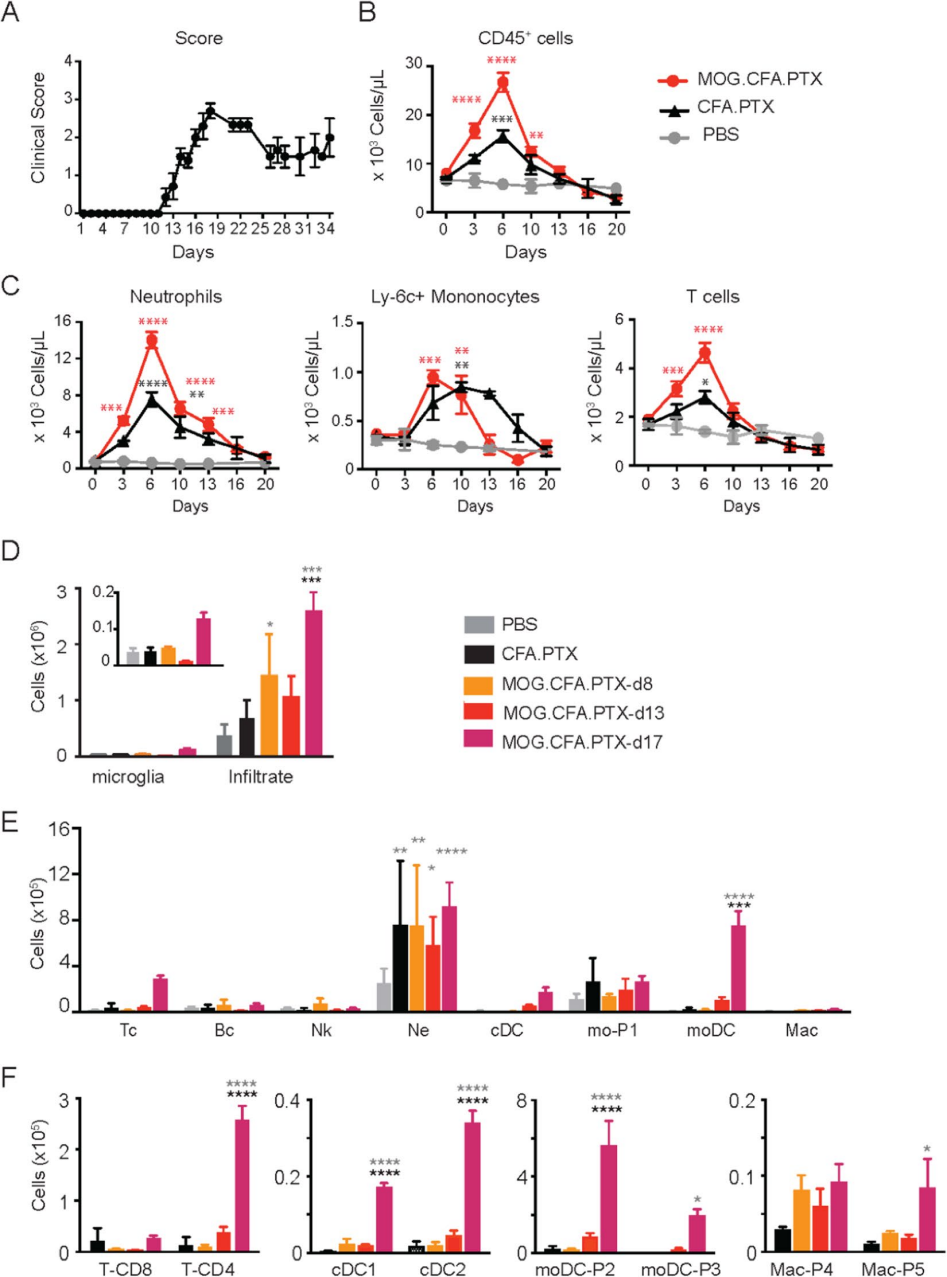
Quantification of blood circulating immune cells and SC infiltrated immune cells during EAE progression. Two way (**A**–**C**) or one way (**D**–**F**) uncorrected ANOVA were used to compare every time point of EAE animals against PBS (grey) or CFA.PTX (black) control conditions. *p < 0.05, **p < 0.01, ***p < 0.005 and ****p < 0.001. (**A**) Clinical scores of EAE mice as a function of time. Clinical score remained null for PBS or CFA.PTX injected mice and are thus not represented. (**B**) Quantification of total blood circulating CD45^+^ cells at different post-EAE induction times. (**C**) Quantification of the number of circulating neutrophils, Ly6C^+^ monocytes and T cells in CD45^+^ cells presented in B. (**D**) Quantification of microglial cells versus infiltrating cells in SC. (inset) Microglia section is zoomed for a better visualization. (**E**) Quantification of the most representative populations of infiltrated cells. (**F**) Quantification of subclasses of T cells, conventional DCs, moDCs and macrophages. Datas are represented as mean value ± SEM. (**A**–**C**) Significant differences between MOG.CFA.PTX and PBS are indicated as red stars and between CFA.PTX and PBS as black stars. (**D–F**) Significant differences between MOG.CFA.PTX and CFA.PTX are indicated as black stars and between CFA.PTX and PBS as grey stars. Populations abbreviations are: T cells (Tc), B cells (Bc), NK cells (NK), neutrophils (Ne), conventional DCs (cDC), monocytes (mo-P1), monocyte-derived DCs (moDC), macrophages (Mac).

We measured clinical scores and the levels of circulating cells in blood of individual mice injected with either PBS, or CFA.PTX or MOG.CFA.PTX, over time (Fig. 2A). Six days after EAE induction, before occurrence of clinical signs, we observed a concentration of CD45^+^ cells in blood twice larger than in CFA.PTX mice (p < 0.001, Fig. 2B). All blood immune cell populations increased, with a strongest rise for neutrophils, T cells and inflammatory Ly6C^+^ monocytes (Fig. 2C). Noteworthy, the number of circulating monocytes quickly decreased to reach a basal level around day 13 shortly after the onset of clinical signs in EAE animals, whereas monocyte levels remained high for 6 additional days in CFA.PTX mice. It is reported that by themselves adjuvant and toxin do not disrupt the BBB or Blood Spinal Cord Barrier (BSCB) shortly after immunization. Under these conditions, neutrophils firmly adhere to the endothelium of meningeal vessels but do not penetrate in the parenchyma^27^. In the CNS of MOG.CFA.PTX (EAE-induced) mice however, a rapid infiltration of these cells was likely responsible for disease onset.

We then compared the number of parenchymal immune cells in SC and brain of these mice (Fig. 2D–F & Supplementary Fig. 1). No changes in cell numbers were observed between day 8 and day 17 in PBS or CFA.PTX control groups, hence data of the different time points were pooled for each condition. Conversely, in EAE-induced mice, the number of cells infiltrating the SC was double at day 8 than in PBS control and four times higher at day 17. Over the same time period, the number of microglia remained stable until a late increase by day 17 (Fig. 2D, inset). Infiltrated peripheral cells thus dominated EAE inflammatory response, and comprised of a large though variable proportion of neutrophils, moDCs, CD4 T cells and comparatively weak infiltrations of cDCs, B and NK cells (Fig. 2E). In the brain, the specific immune response is similar to the SC, except for earlier accumulation of T cells and a more modest recruitment of neutrophils (Supplementary Fig. 1).

At the peak of the disease (day 17) the infiltrate of immune cells was at its maximum both in the SC and brain. T cells were mainly of CD4^+^ phenotype, while dendritic population was overwhelmingly represented by moDCs (P2 and P3) compared to the small number of cDC cells (Fig. 2E & Supplementary Fig. 1). moDCs represented 24 to 31% of total immune cells and, at day 17, exceeded microglia counts by 3–4 folds in the SC. The macrophage population (P4 and P5) remained low at all time points, with an increase of the more mature P5 MHCII^hi^ differentiation stage at day 17 (Fig. 2F & Supplementary Fig. 1).

### Transgenic fluorescent mouse model permit visualization of identified immune cell subpopulations

**Figure 3:**
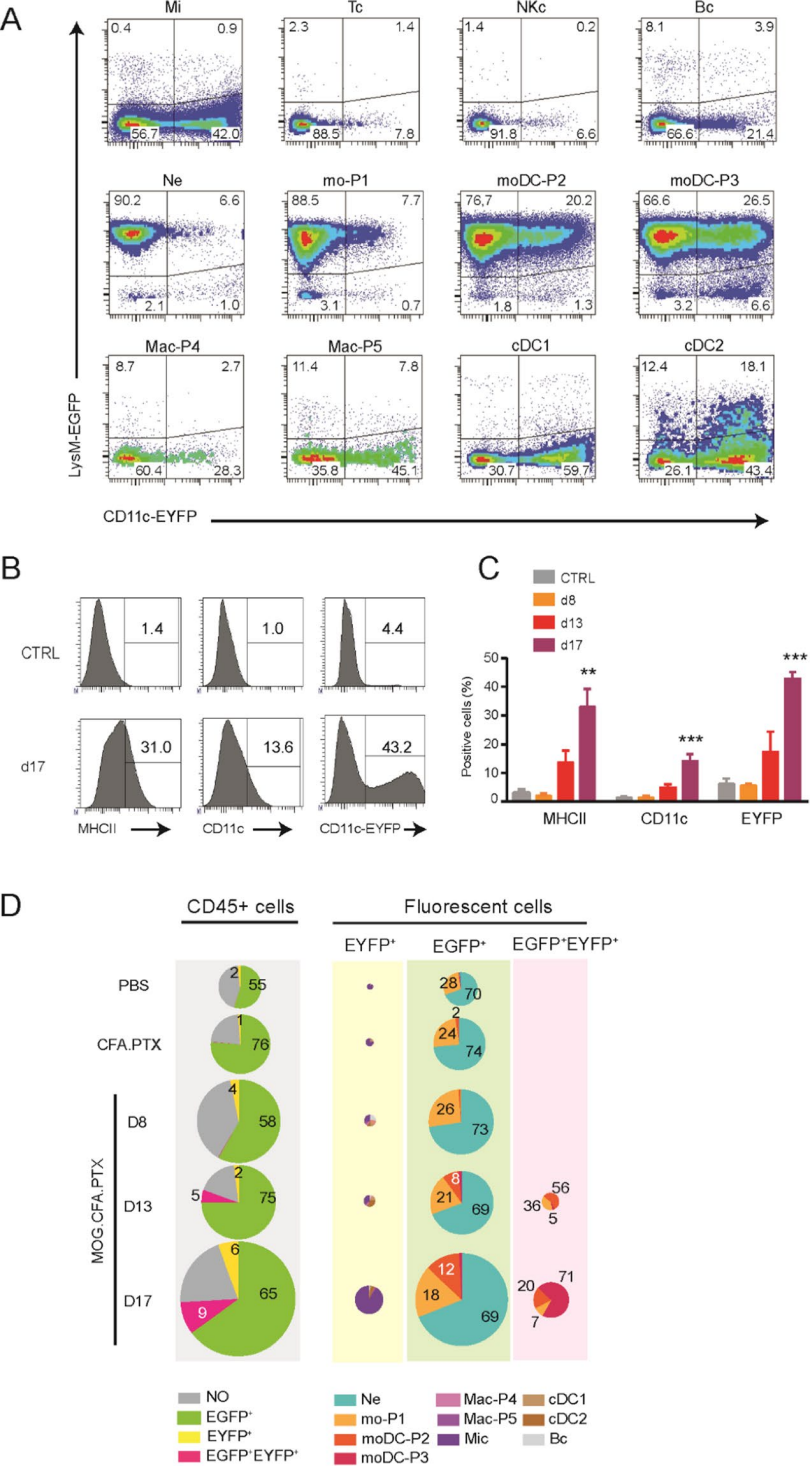
Characterization of the fluorescently labeled immune cells in Thy1-CFP//LysM-EGFP//CD11c-EYFP mice. (**A**) LysM-EGFP and CD11c-EYFP expression in microglia and the various immune cell types infiltrating the CNS during EAE progression. Multicolor contour plots for immune cells extracted from brain at day 17: Microglia (Mi), T cells (Tc), NK cells (NKc), B cells (**B**), neutrophils (Ne) monocytes (mo-P1), moDCs (moDC-P2 and moDC-P3), macrophages (Mac-P4 and Mac-P5), conventional DC (cDC1 and cDC2). **(B)** Distribution histograms for microglia cells of control (CTRL) and at day 17 (d17) SCs. **(C)** Bar graph showing the expression of MHC class II, CD11c and CD11c-EYFP in microglia cells of control (CTRL) and day 17 (d17) SCs. The percentages of positive cells are indicated on the histograms and their evolution is presented as bar graphs. One way uncorrected ANOVA was used to compare every time point of EAE animals against PBS (grey) or CFA.PTX (black) control conditions. *p < 0.05, **p < 0.01, ***p < 0.005 and ******p < 0.001. **(D)** Quantification and distribution of fluorescent cells during EAE in SC. In the first column, the distribution of CD45^+^ cells in EGFP^+^, EYFP^+^ or EGFP^+^/EYFP^+^ fluorescence channel is represented for control (PBS or CFA.PTX) or induced (MOG.CFA.PTX) mice at day 8, 13 or 17. The 3 columns on the right show the relative numbers of fluorescence cells and their characterization using the gating strategy. The pie chart sizes are proportional to the total number of cells. The numbers indicate the percentage of the corresponding population.

moDCs are a poorly documented population of cells in the context of EAE due in part to their complex identification. We previously showed that moDCs co-express EGFP and EYFP in LysM-EGFP//CD11c-EYFP mice^21^. We induced EAE in Thy1-CFP//LysM-EGFP//CD11c-EYFP triple heterozygous transgenic mice and provided a detailed description of the fluorescence protein expression among the previously identified subpopulations (Fig. 3 & Supplementary Fig. 2). LysM-EGFP transgene labeled neutrophils, monocytes and moDCs. Noteworthy 20% of EGFP^+^ moDC-P2 and 30% of EGFP^+^ moDC-P3 also co-expressed the CD11c-EYFP transgene, suggesting a maturation-dependent expression of EYFP. Although CD11c-EYFP transgene was expressed alone in 20% of total B cells and up to 50% of total cDCs or macrophages (Fig. 3A), these populations were small and therefore had virtually no contribution to the EYFP^+^ population at peak response (Fig. 3D). The large majority of CD11c-EYFP^+^/LysM-EGFP^−^ cells was identified as activated microglia whose number increased with disease progression concomitantly with increased expression of activation markers in the total microglia population (CD11c, MHCII; Fig. 3B,C). Yet the percentage of fluorescent cells did not vary significantly with disease progression in any of the predefined populations, except for microglia (Fig. 3B,C).

In SC, (Fig. 3D) in all conditions, over 55% of the CD45^+^ population was EGFP^+^ whereas EYFP^+^ cells were a minority (<6%). These EGFP^+^ cells were identified mainly as neutrophils (~70%) and monocytes (~30%). Nearly all of these monocytes were P1 before day 8. The smaller EYFP^+^ population was identified as densely labeled microglia (>90% of EYFP^+^ cells at day 17). A population of EGFP^+^/EYFP^+^ double-labeled moDCs (moDCs P2 and P3) appeared at day 13 and expanded into a mature moDCs population composed of 70% moDC-P3 by day 17.

In the brain (Supplementary Fig. 2), the immune response was very similar to the SC except for the smaller percentage of fluorescent cells in the CD45^+^ population due to a reduced infiltration of neutrophils and an increased percentage of microglial cells compared to SC.

### Spatio-temporal distribution of fluorescent cells in SC

**Figure 4:**
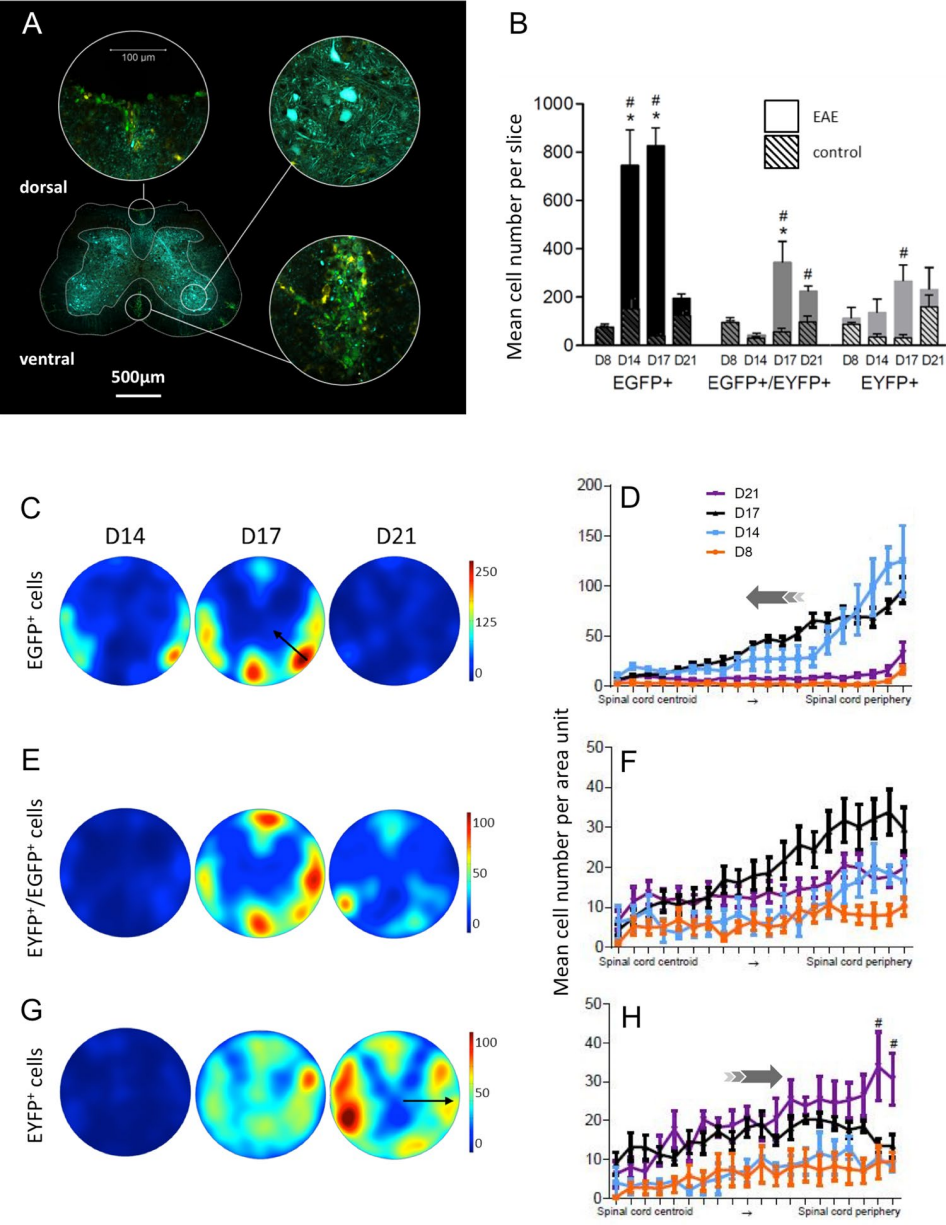
Dorso-ventral localization of EYFP^+^ and EGFP^+^ cells in coronal SC slices. (**A**) Infiltration of EGFP^+^ and EYFP^+^ cells in the SC of a triple fluorescent EAE mouse at day 17 post-induction. Edges of the slice were highlighted with dotted lines. Scale bar 500 µm. Zoomed images in white matter plaques or grey matter regions. Green: EGFP^+^ cells. Yellow: EYFP^+^ cells. Cyan: CFP^+^ neurons. **(B)** Bar graphs showing EGFP^+^, EYFP^+^ and EGFP^+^/EYFP^+^ cell quantification in SC slices from EAE (Plain) and CFA.PTX control (Hatched) mice. Black: EGFP^+^ cells. Grey: EGFP^+^/EYFP^+^ cells. Light grey: EYFP^+^ cells. Star: significant evolution compared to day 8 within a group of subjects. Sharp sign: intergroup significance at a given time point (n = 3–4). EGFP cell number is higher in EAE SC compared to control at both D14 (Non-parametric Mann-Whitney test: p = 0.016) and D17 (Non-parametric Mann-Whitney test: p = 0.029). EYFP cell number is higher in EAE SC compared to control at D17 (Non-parametric Mann-Whitney test: p = 0.03). EYFP/EGFP cell number is higher in EAE SC compared to control at D17 (Non-parametric Mann-Whitney test: p = 0.016) and D21 (Non-parametric Mann-Whitney test: p = 0.029). **(C**,**E**,**G)**. Color coded average maps of cell densities in SC slices (n = 3–5 for each time point and condition) at different post induction days (**D**). **(D**,**F**,**H)** Distribution of cells density from the center to the periphery of the density maps over time. Average number of cells present in each concentric circular band of the map, for all SC slices (see supplementary methods & figures). Gray arrows show the direction of cells progression: (**D**) from the periphery to the center; (**H**) from the center to the periphery. (**C**,**D**) EGFP^+^ cells. (**E**,**F)** EGFP^+^/EYFP^+^ cells. (**G**,**H**) EYFP^+^ cells. EGFP cells are more numerous at the periphery compared to center of slices at both D14, D17 (Non-parametric Kruskal-Wallis test: p < 0.0001) and D21 (Non-parametric Kruskal-Wallis test: p = 0.01). EGFP/EYFP cells are more numerous at the periphery compared to center of slices at both D17 and D21 (Non-parametric Kruskal-Wallis test: p < 0.0001). EYFP cells are less numerous at the periphery at D17 compared to D21 (Non-parametric Mann-Whitney test: p = 0.0002).

To characterize the interactions between fluorescent immune cells and neurons at the cellular level, as well as their distribution pattern during pathology progression, we acquired confocal microscopy images of fixed SC slices from Thy1-CFP//LysM-EGFP//CD11c-EYFP mice taken at days 8, 14, 17 and 21 after EAE induction. At days 14 and 17, the density of fluorescent cells was strikingly higher in EAE-induced than in control CFA.PTX mice (Fig. 4A,B & Supplementary Fig. 3). We observed large numbers of EGFP^+^ cells at days 14 and 17 as well as the delayed accumulation of double-labeled EGFP^+^/EYFP^+^ cells. We also highlighted patches of fluorescent cells in plaques, only in white matter tracts. No EGFP^+^ cell was observed in the grey matter, characterized by a high density of CFP^+^ neuronal cell bodies and neurites (Fig. 4A**)**. Noteworthy, plaques were more numerous in the ventro-lateral than in the dorsal part of the hemicord (4 versus 1 plaque/slice). At each time point examined, the density of EYFP^+^ cells was not significantly different from CFA.PTX control except at day 17 where the density of EYFP^+^ cells doubled (Fig. 4B). These cells were mainly located in plaques although some isolated cells could be observed in both white and grey matter (Fig. 4A).

To take into account the uneven distribution of plaques across SC, we evaluated the average densities of cells by summing the results obtained from several slices with normalized polar coordinates. Cell distributions were established at all distances from SC center and color-coded in arbitrary units at different time points to access their dynamics (see methods) (Fig. 4C–H). The data revealed that EGFP^+^ cell infiltration occurs from the peripheral edges at day 14, preferentially, though not exclusively, from the motor ventro-lateral funiculi. Infiltrated cells then accumulate deeper in the tissue as indicated by the dampening of peripheral densities and coincident increase of densities deeper in the parenchyma observed at day 17 (black arrows on Fig. 4C,D). Infiltration of white matter and cell aggregation in plaques also occurred in the dorsal white matter along a similar timeline. Strikingly, at day 21, a lower number of cells remained in plaques (Fig. 4C,D).

EGFP^+^/EYFP^+^ cells were sparse at day 14, but their numbers increased dramatically to a peak density at day 17. These cells were located in white matter areas where EGFP^+^ cells were already present (Fig. 4E,F), suggesting that monocytes P1 (EGFP^+^) mature into moDCs P2-P3 (EGFP^+^/EYFP^+^) *in situ*. At day 21, the majority of EYFP^+^/EGFP^+^ cells had disappeared, and the remaining cells were grouped in residual plaques spread in white matter (Fig. 4E,F).

Similar to EYFP^+^/EGFP^+^ cells, EYFP^+^ cells density peaked at day 17 but were distributed, across the entire SC, including the white and grey matters. At day 21 however, EYFP^+^ cells were significantly more located in the white matter (Fig. 4G,H black arrows), preferentially in plaques.

**Figure 5:**
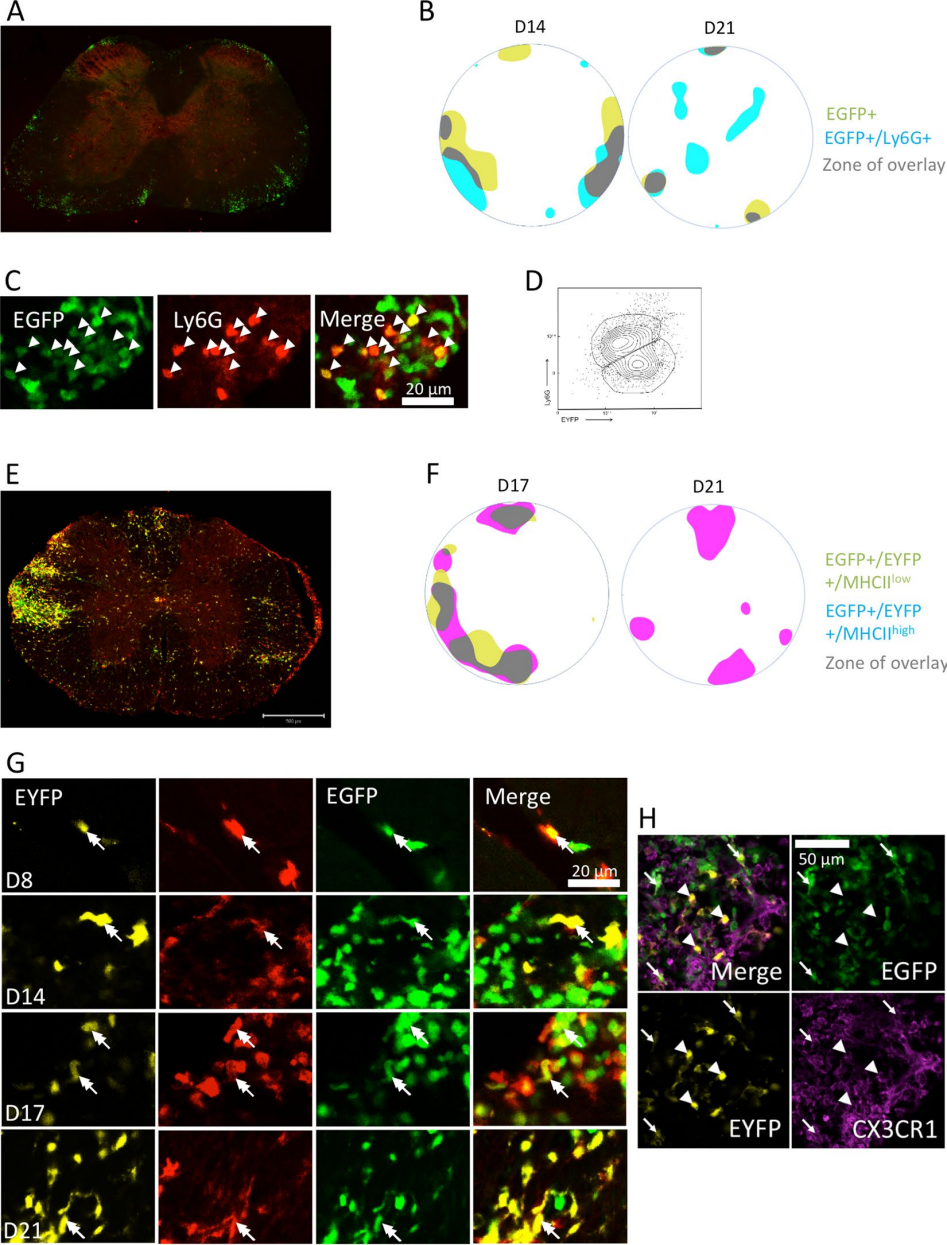
Patterned distribution of infiltrated immune cells in SC slices. (**A**) Ly6G^+^ immunostained coronal slice of a LysM-EGFP^+^/CD11c-EYFP^+^ SC 14 days after EAE induction. EGFP^+^ cells (green) and Ly6G^+^ (red). (**B**) Average density maps of EGFP+ (yellow) and EGFP+/Ly6G+ (blue) cell 14 and 21 days after EAE induction. Regions of overlap are shown in grey. (**C**) High magnification image inside a plaque 17 days post-induction. EGFP+ cells (green) and Ly6G+ (red). EGFP+//Ly6G+ are neutrophils (arrow heads) and EGFP+/Ly6G− monocytes-P1. Note that all Ly6G+ cells are also EGFP+ (arrow heads). **(D)** Cluster representation of the population of EGFP^+^ cells detected in the slices, according to their expression of Ly6G^+^, 14 days after EAE induction. **(E)** MHCII^+^ immunostained SC coronal slice of a LysM-EGFP//CD11c-EYFP mouse 17 days after EAE induction. EYFP^+^ cells (yellow), EGFP^+^ cells (green), MHCII labeling (red). Scale bar: 200 µm. **(F)** Average density maps of EGFP+/EYFP+/MHCIIlow (yellow) and EGFP+/EYFP+/MHCIIhigh (pink) cells 17 and 21 days after EAE induction. Regions of overlaps are shown in grey. **(G)** High resolution image of a plaque showing a MHCII^+^/EGFP^+^/EYFP^+^ cell (double-headed arrow) Scale bar: 20 µm. **(H)** High resolution image of a plaque at D21 showing EYFP^+^/EGFP^−^/CX3CR1^+^ (microglia; arrow heads) and EYFP^+^/EGFP^+^/CX3CR1^+^ (moDC; arrows) cells. Scale bar 50 µm.

To further refine our description of fluorescent inflammatory cells, we performed immunostainings for Ly6G, MHCII and CX3CR1 from tissue collected at 8, 14, 17 and 21 days post-EAE induction (Fig. 5 & Supplementary Fig. 3). Ly6G allowed identifying two phenotypes among EGFP^+^ cells (Fig. 5A–D): Ly6G^+^ neutrophils and Ly6G^–^ monocytes. Approximately 65% of EGFP^+^ cells were Ly6G^+^ at day 14 and 60% at day 17. These ratios were comparable to those found in flow cytometry experiments (Fig. 3D, 70% of neutrophils versus 30% of monocytes P1). Interestingly, at day 14, comparing the average densities of EGFP^+^/Ly6G^+^ and EGFP^+^/Ly6G^−^ cells revealed that they were overlapping is some regions, but not perfectly superimposed, with more neutrophils (Ly6G^+^) in the ventral area and more monocytes (Ly6G^−^) in lateral areas (Fig. 5B). This observation was consistent with both the expected initiating role of neutrophils in the innate inflammatory cascade and the ventral occurrence of the earliest inflammatory events in EAE. CX3CR1 was expressed by more than 90% of EYFP^+^ cells and ~50% of the EGFP^+^/EYFP^+^ cells at day 21 (Fig. 5H). MHCII, an activation/maturation marker, also allowed identifying two phenotypes among EGFP^+^/EYFP^+^ cells corresponding to MHCII^lo^ and MHCII^hi^ subsets (Fig. 5E,G). At day 17, ~70% of EGFP^+^/EYFP^+^ cells were MHCII^hi^ (Fig. 5F). Numerous non-EGFP and non-EYFP MHCII^+^ cells were also observed. At day 8, these cells were located in the meningeal area, while at post-induction days 14, 17 and 21, they entered the edges of the SC through the white matter. These cells could be B cells as indicated by MHCII gating used in cytometry experiments, or non-fluorescent cDCs.

### *In vivo* imaging of the dynamics and morphologies of fluorescent immune cells in the dorsal white matter

**Figure 6:**
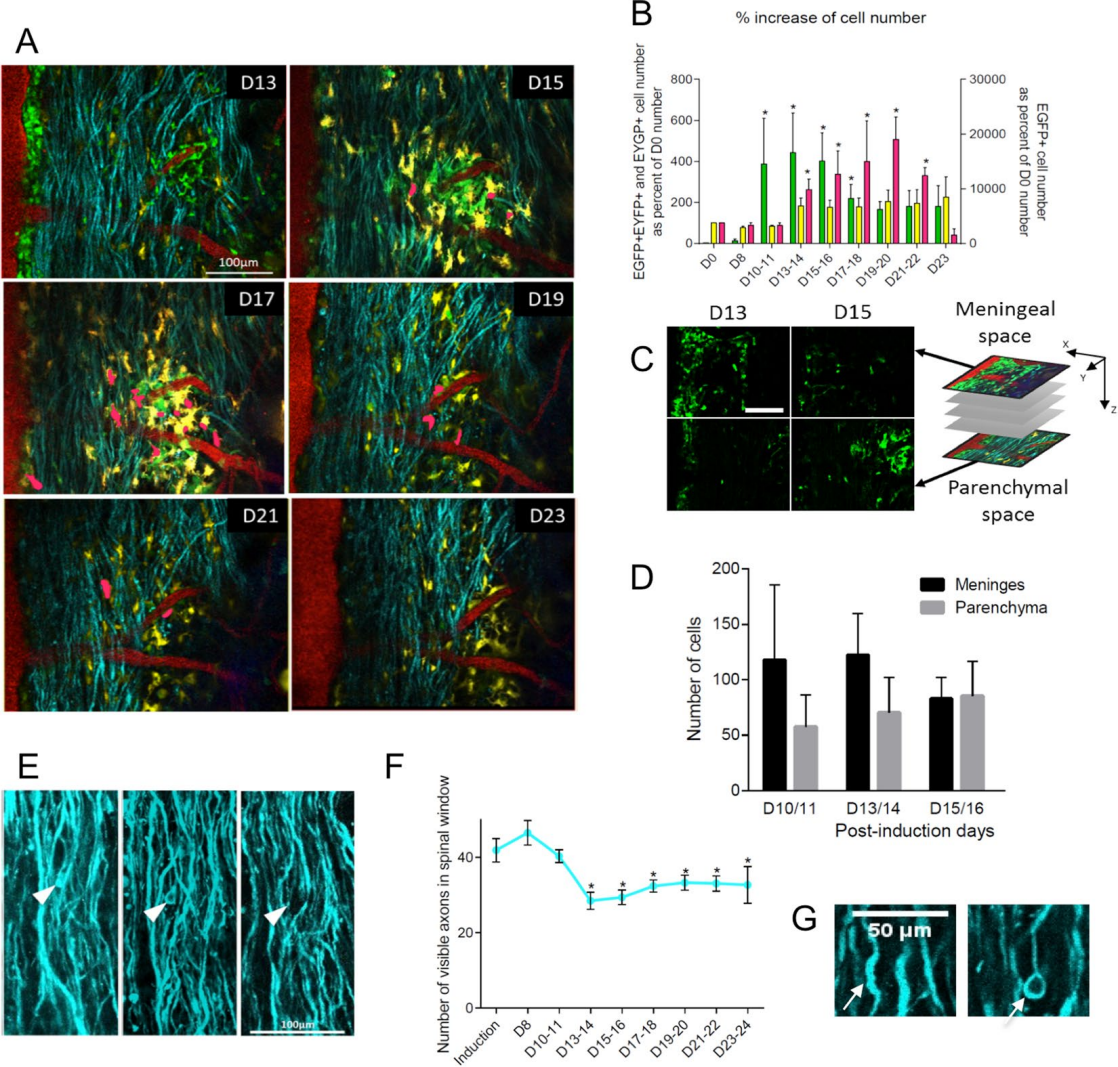
*In vivo* visualization of immune cell dynamics during EAE progression. EGFP^+^ cells (green), EYFP^+^ cells (yellow), EGFP^+^/EYFP^+^ cells (pink, manually highlighted), CFP^+^ neurons (cyan), blood vessels (red), type 2 collagen (dura mater, Blue). D = post-induction day. **(A)** Dynamics of cell distribution during EAE at different post-induction days. Scale bar: 100 µm. **(B)** Evolution of immune cell densities relative to pre-induction values. EGFP+ cells (green), EYFP+ cells (yellow), EGFP+/EYFP+ cells (pink). EGFP cell number changes during EAE (Non-parametric Kruskal-Wallis test: p = 0.001), starting at D10-11 (Non-parametric Mann-Whitney test: p = 0.029). EGFP/EYFP cell number changes during EAE (Non-parametric Kruskal-Wallis test: p = 0.005), starting at D13-14 (Non-parametric Mann-Whitney test: p = 0.044). EYFP cell number does significantly change during EAE (Non-parametric Kruskal-Wallis test: p = 0.635). (n = 9, *p < 0.05 compared to induction). **(C)** Depth-dependent gradient of EGFP^+^ cells at D13 and D15. Note the initial meningeal accumulation of cells and subsequent infiltration into the tissue. Scale bar 100 µm. **(D)** Quantification of EGFP^+^ cells in meningeal and deep parenchyma areas at days post-induction 10/11, 13/14 and 15/16. Black bars: meningeal area. Grey bars: parenchyma area. **(E)** Evolution of identified individual axons at different post-induction days. Arrow head points to a damaged axon. Scale bar 100 µm. **(F)** Evolution of the number of CFP^+^ axons in determined regions of interest at different stages of EAE progression. Axon number evolves during EAE (Non-parametric Kruskal-Wallis test: p = 0.001), starting at D13-14 (Non-parametric Mann-Whitney test: p = 0.031). (n = 9, *p < 0.05 relative to pre-induction values). **(G**) Axonal damages: swelling, without (left) or with (right) disconnection of the distal segment. Scale bar 50 µm

In order to better characterize the dynamics of recruitment and maturation of these cell populations inside the SC, we performed recurrent imaging of individual mice at days 0, 8, 10, 13, 15, 17, 21, 23 after induction (Fig. 6). Micrometric repositioning of the animal as well as post-acquisition spatial registration of the images allowed for repeated observation and quantification of cell densities and distributions in the same region of interest throughout disease progression in every animal. Supplementary Fig. 4A illustrates the projection image of an in depth wide field of the dorsal white matter track.

In line with our histological data, we observed a transient but massive infiltration of EGFP^+^ cells that started at day 10–11, before clinical disease onset (Fig. 6A,B). EGFP^+^ cell densities peaked at day 13, with a subsequent decline in numbers that was inversely correlated with the occurrence of EGFP^+^/EYFP^+^ cells, whose density was maximal on days 17–20. Though statistically non significant, a slight and progressive increase in EYFP^+^ cell densities was observed along disease progression. Only a few EGFP^+^/EYFP^+^ cells could be clearly identified in the intravital images because of the optical properties of living tissue.

Dynamic images of living tissue highlighted a depth-dependent distribution of inflammatory cells with a top down gradient of EGFP^+^ cells coming from meninges (Fig. 6C,D) as suggested by our data on tissue sections. Several EGFP^+^ cells could be seen circulating in blood vessels (Supplementary Video 1) at all studied post-induction days, either quickly moving in blood flux or slowly rolling along the internal wall of the blood vessels. Such rolling cells have been reported as ready to undergo extravasation^28^, however despite long periods of observation (>5 h/imaging session) we did not observe any extravasation events. By contrast, many EGFP^+^ cells could be seen closely associated with, and migrating along, the external surface of blood vessels especially at early stages (Supplementary Video 1 & 4). We therefore concluded that EGFP^+^ cells invade the white matter from peripheral meninges to deep spinal parenchyma by following perivascular spaces.

We then performed quantitative analyses of axons densities and blood vessels diameters over time at a fixed intermediate depth in tissue suitable for both axons and EGFP^+^ cells vizualisation. Recurrent measurements of blood vessel diameters (Supplementary Fig. 4B) revealed a progressive dilation, that became significant on day 10, concomittant with the peak of infiltrating EGFP^+^ cells. Vessel dilation then persisted throughout the disease. Noteworthy, fluorescent axons counted in a given region of the SC dropped significantly on day 13 (Fig. 6F, Supplementary Fig. 5), immediately after the massive infiltration of EGFP^+^ cells. It stabilized thereafter, concomitantly with the decline of EGFP^+^ cells densities and the coincident maturation of monocytes P1 into moDCs P3 and accumulation of EYFP. This reduction in axon counts in plaques was explained by a progressive loss of axon integrity, subsequent swelling and degeneration (Fig. 6E–G), and was not observed in areas free from immune cells (Supplementary Fig. 5). Clinical scores were evaluated on the animal before each imaging session, and were significantly correlated with axonal densities.

**Figure 7:**
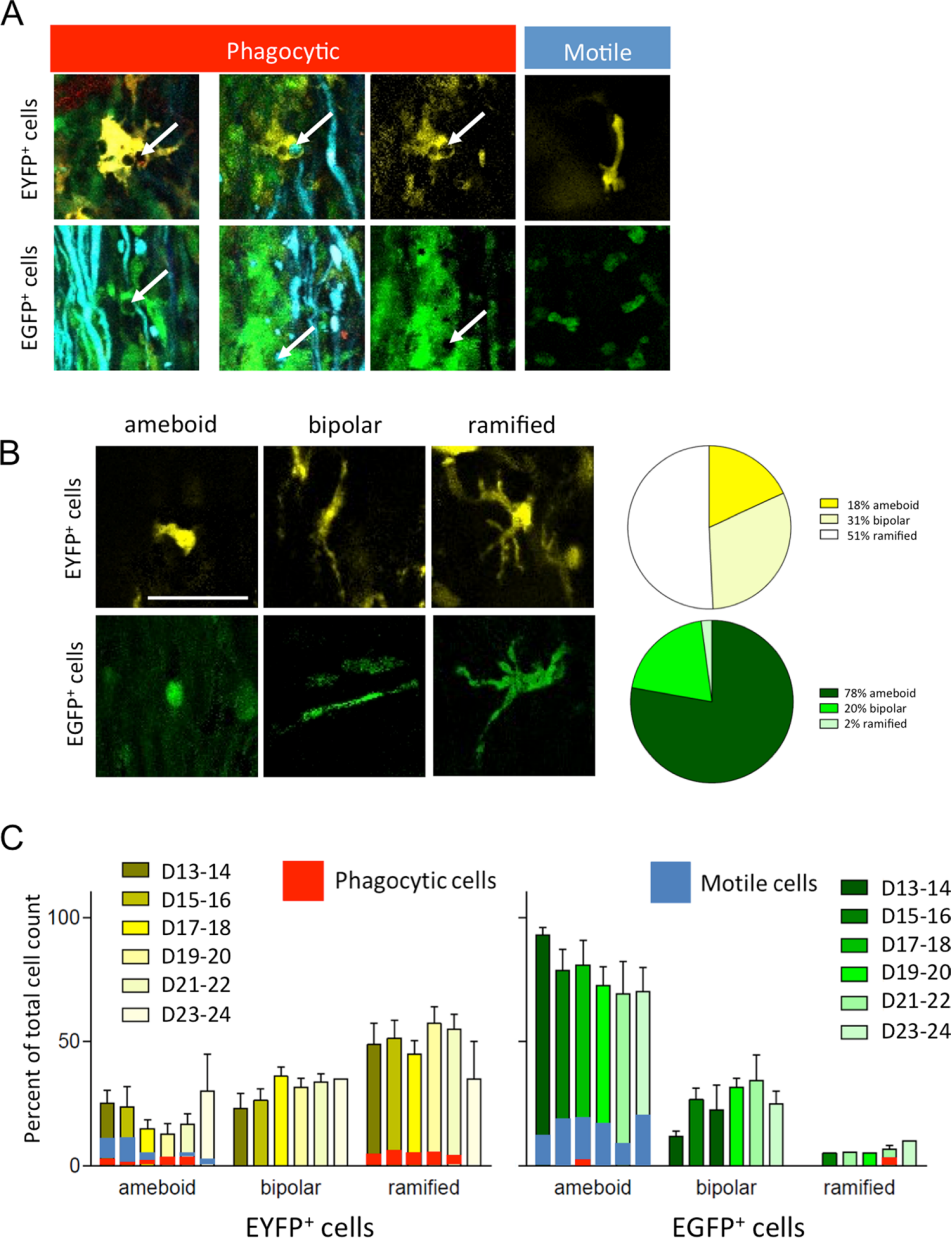
*In vivo* characterization of neuroimmune interactions. (**A**) Phagocytosis of axon debris by EYFP^+^ and EGFP^+^ cells. Phagocytic activity is associated with ramified morphology while motile cells that are non-phagocytic present elongated morphologies (n = 5). Middle panel presents phagocytic EYFP^+^ and EGFP^+^ cells either in their multicolor cellular environnement (left) or as a single channel image (right). Arrow point to phagocytic bodies. Scale bar 20 µm. (**B**) Quantification of the three main classes of morphologies for EGFP^+^ and EYFP^+^ cells: ameboid, elongated, ramified. **(C)** Evolution of the distributions of EGFP^+^ and EYFP^+^ cell morphologies during the course of the pathology (n = 5, D = post-induction day. *p < 0.05). The proportion of phagocytic (red) and motile (blue) cells is overlaid on the bar graphs.

Then we examined the *in vivo* images to look for evidence of subcellular interactions between EGFP^+^ and EYFP^+^ immune cells and axons with a focus on phagocytosis. Throughout the disease progression, rare phagocytic events were observed in EGFP^+^ cells whereas these were observed in 15% the EYFP^+^ population (Fig. 7A–C). Due to the low number of double-labeled EGFP^+^/EYFP^+^ cells detected under our *in vivo* imaging conditions, trustable quantitative analyses could not be conducted on them.

Next we explored the evolution of fluorescent cell morphologies and motility. Approximately 80% of EGFP^+^ cells were small and ameboid (Supplementary video 3), ~20% bipolar (Supplementary video 4), and very few (~2%) were ramified (Supplementary video 5). The bipolar cells were mainly observed along blood vessels (see above and Supplementary video 1 and 4) or along axons parallel to the rostro-caudal axis (Fig. 7B & Supplementary Fig. 5). Ameboid cells were highly motile (Fig. 7C & Supplementary video 2 and 3) and tend to decrease on days 19–20 compared to day 13–14, concomitantly with a peak in bipolar cells (Fig. 7C). Quantitative analysis of cell morphologies pointed out that the solidity of EGFP^+^ cells rather decreased from the day of disease onset until day 23. Although non statistically significant, this decrease is consistent with a progressive shift of ameboid shapes toward ramified ones as monocytes maturate into APC (Supplementary Fig. 6B). Similarly eccentricity measurements revealed a two phases process that reached statistical significance at the time of lowest solidity of the cells (Supplementary Fig. 6C). 50% percent of the EYFP^+^ cells were ramified (Fig. 7A), and real-time changes in their shapes were observed as processes probed the local environment (Supplementary videos 2 & 5). Phagocytic inclusions could be observed in approximately 15% of both ameboid and ramified populations (Fig. 7A,C). A non-significant decrease of EYFP^+^ ramified cells was observed by day 23–24 in favor of more ameboid morphologies. In control CFA.PTX mice EYFP^+^ cells remained mainly ramified without noticeable changes over time (Supplementary Fig. 6D). Thus the different types of morphologies observed in EAE-induced animals (Fig. 7B & Supplementary Fig. 6D) revealed that the different states of EYFP^+^ cells activation are specifically triggered by immunization against the MOG peptide.

**Figure 8:**
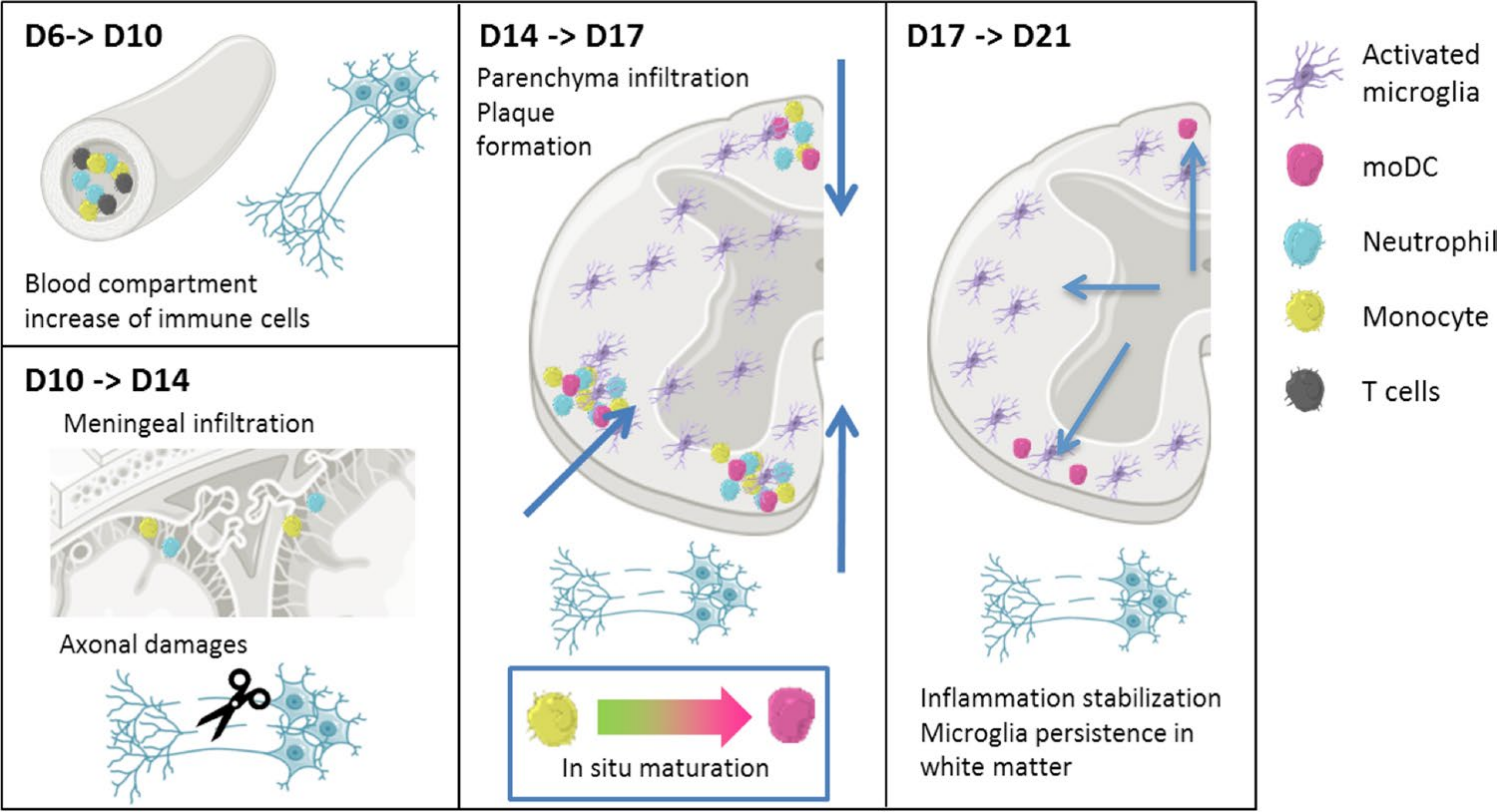
Schematic representation of dynamic events occuring in EAE disease. (1) Activation of blood vessels during diapedesis of MOG specific CD4+ cells followed by accumulation of circulating immune cells and the weakening of BSCB; (2) susbequent infiltration of EGFP+ neutrophils and P1 monocytes via the meninges that is concomitant with axonal losses in the white matter (scissors). (3) This infiltrate would activate microglia, involved both in shaping the molecular environment and phagocytosis of the axon debris all over the spinal cord. Evolution of the chemical cues between D14 and D17 would be responsible for differentiation/maturation of monocytes into EGFP+/EYFP+ moDCs during their migration deep into the tissue. (4) Their APC phenotype would subsequently trigger a selective antigen specific immune response while dampening microglial activation. Activated microglia accumulate in peripheral white matter plaques and recover a resting phenotype by D21, while moDCs disappear. Figure was made using Servier Medical Art under Creative Commons Attribution 3.0 Unported License https://creativecommons.org/licenses/by/3.0/, no changes have been made to the material.

Altogether, cytometry, *in vivo* imaging and immunostaining experiments demonstrate that recruitment of innate immune cells follows a complex sequence of events, involving different patterns of activation and various cell types. In particular we demonstrated that an initial fast rise in EGFP^+^/EYFP^−^ monocytes P1 at the end of the second week followed by a rise in EGFP^+^/EYFP^+^ moDCs in the third week post induction signed the phenotypic evolution of the immune response in relation with a stabilization of clinical scores (Fig. 8).

## Discussion

EAE pathology is driven by MOG-reactive Th1 and Th17 CD4 T cells, that are reactivated by APCs in perivascular area of CNS and initiate an inflammatory cascade resulting in massive recruitment of leucocytes^29^. Defining the phenotypes and distinct dynamics of recruited innate cell subsets is crucial to characterize the interplay between innate and adaptive immune systems as well as its impact on neurons and myelin. In this study, we designed a strategy to precisely follow axonal loss and discriminate over time the main components of innate immune response in Thy1-CFP//LysM-EGFP//CD11c-EYFP EAE mice.

Our transgenic markers combined with polychromatic antibody panels and corresponding flow cytometry gating strategies allowed us to observe, for the first time, the densities of 12 cell subsets in individual EAE-induced mouse brains and SCs at different stages of disease. The strategy used is based on recent work done in the skin^15^ where CCR2 surface receptor was used in combination with CD64 to discriminate monocyte-derived DCs from macrophages. Although useful in healthy CNS, these markers became useless under inflammatory conditions. We showed that combination of CD44 and CD64 improved the gating strategy. Although this paradigm alone was unable to differentiate the recently described long-lived non-parenchymal macrophages of embryonic origin^12^, we showed in a chimeric model that we could overlook this population that represented less than 10% of the CD44^−^ macrophages and did not increase upon EAE-induced inflammation. Our study is thus complementary and brings additional data to those obtained with studies on CNS inflammation^12,14,30^.

The LysM-EGFP//CD11c-EYFP mice proved useful to label 50 to 80% of all infiltrated immune cells during disease, among which the most critical populations were neutrophils, monocytes (P1), moDCs (P2, P3 maturation states), macrophages (P4, P5 maturation states) as well as microglia. Whereas the earlier reporter mouse model CCR2-RFP//CX3CR1-GFP is a powerful tool to study the dynamics of innate immune cells during CNS inflammation, we consider our study complementary. In the CCR2-RFP//CX3CR1-GFP reporter mouse, all CNS resident macrophages including microglia, perivascular, choroid plexus and meningeal macrophages are CX3CR1-GFP^high^^12,14,30,31^; in our model these cells are LysM-EGFP^−^ and microglia express CD11c-EYFP when activated. CCR2-RFP labeled strongly inflammatory monocytes; in our model these cells are LysM-EGFP^+^, they evolved to CCR2^+^ moDCs that expressed both LysM-EGFP and CD11c-EYFP. These observations provide evidence that CCR2 is not a specific marker of moDCs in inflammatory conditions. Through recurrent observations by two-photon microscopy we additionally got access to the histomorphology, 3D spatiotemporal distribution and *in situ* dynamics of these cells throughout disease progression and corresponding motor deficits.

Infiltration of neutrophils was significant in both the brain and SC, but more modest in the brain, likely due to increased vulnerability of the BSCB over BBB^32^. Although cytometry data showed high densities of neutrophils in SC as early as day 8 both in EAE-induced mice and in CFA.PTX controls, these data were not confirmed using immunohistochemistry or *in vivo* imaging techniques. One possible explanation for this is that MOG immunization was required to trigger the infiltration of blood circulating neutrophils and monocytes, raising the possibility that the dissociated cell samples were contaminated by meningeal and endothelium adherent cells^27^ whose density was highest at this time point. Nevertherless at all time points, we observed an excess of infiltrated Ly-6G^+^ neutrophils over infiltrated monocytes in SC. Because neutrophils can release molecules that can harm neurons^33^, reduction in neuronal losses can be obtained by limiting their recruitment^27,34^. Similarly infiltrated monocytes can affect disease progression by either dampening^35^ or enhancing^36^ T cells response according to chemical environment.

The route of leucocyte infiltration into the CNS remains controversial. Two entry routes have been described in MS models, either via the choroid plexus and the leptomeningeal vessels, or by the parenchymal capillaries^37^. Proinflammatory monocytes were shown to enter SC through adjacent leptomeninges after injury^38^. In our study, EGFP^+^ cell extravasation from vessels, as previously reported^27^, was not observed during *in vivo* observations. Instead, accumulation of EGFP^+^ cells in meninges disappeared coincidently with a perivascular accumulation of these cells along vessels or along axons, supporting the idea that EGFP^+^ cells first entered the SC parenchyma through meninges rather than by extravasation. Similar to T cells^28^, the EGFP^+^ cells in our study, might have been rapidly guided to inflamed areas by intraparenchymal cerebro-spinal fluid fluxes and were subsequently stopped at sites by chemical cues provided by local cells such as astrocytes, microglia or other infiltrated immune cells. The chemical environment was finally responsible for the differentiation of infiltrated EGFP^+^ monocytes into MHCII^+^ APCs, namely moDCs^15^ as attested by their progressive expression of EYFP once inside the parenchyma. moDCs have been described as short-lived inflammatory cells^39^. They are known to remain mainly in tissue where they develop and to contribute to local immune response. They secrete pro-inflammatory molecules (TNFβ and iNOS) and promote helper T cell polarization^39^. Activation of the TGFβ receptor selectively on these cell can regulate their pro-inflammatory action and is required to trigger remission phase of the EAE disease^40^. Further experiments using mice depleted of these populations should help clarifying their roles.

Resident microglia are an important contributor to the local chemical environment, releasing either prohealing growth factors or inflammatory cytokines depending on their activation states^41^. Although expression of CD11c-EYFP had been reported in many cell types including cDC and macrophages^42,43^, cytometry data indicated that in this study more than 90% of EYFP^+^ cells were microglia in the SC of EAE mice 17 days after induction. EYFP^+^ microglia were the latest cells to accumulate in the diseased SC parenchyma. Despite their increase at day 17, EYFP^+^ microglia remained dispersed throughout the SC, including in grey matter. In following days, their parenchymal distribution shifted towards plaques in the external part of the white mater where they remained until at least day 23. The activated state of these EYFP^+^ microglia was confirmed by the expression of both CD11c and MHCII and the presence of phagocytic bodies in ~15% of these cells. However, the morphological differences of EYFP^+^ microglia between CFA.PTX control and EAE animals indicates morphologies reflect different states of activation. Moreover these morphologies coexisted at the same time in the same animal. Whether morphological phenotypes can be correlated with demyelinating^44^ or promyelinating phenotype^45,46^ still remains to be determined. Physical interactions with infiltrated neutrophils or monocytes were unlikely responsible for activation of microglia into EYFP^+^ microglia given their respective peripheral versus dispersed distributions, suggesting involvement of diffusible stimuli, including antibodies and complement fragments^47^. Moreover, density of double-labeled moDCs declined before the peak of EYFP^+^ microglia suggesting the existence of a regulatory mechanism for mutual exclusion of these cells populations. Competing and opposing roles of microglia and monocyte-derived cells in EAE progression is indeed further illustrated by the demonstration that TNFR2 receptor triggers either a deleterious or protective immune response depending on whether it is activated on monocytes or on microglia^13^.

Having shown that LysM-EGFP//CD11c-EYFP expression can be used to uniquely differenciate moDCs from macrophages as well as to visualize the maturation of some innate immune cells in EAE mice in real-time throughout disease progression, our study set a framework to understand the roles of different subpopulations of myelomonocytic and microglial cells. It will be important for the development of therapeutic strategies that target specific immune cell populations at precise times of disease progression.

## Material and Methods

### Animals

#### Generation of bone marrow chimeras

Seven- to 8-week-old B6-CD45.1 mice were conditioned by treatment with the chemotherapeutic agent Busulfan^7^. Treated mice were reconstituted by i.v. injection of 2.10^6^ bone marrow cells isolated from femurs and tibias of adult B6-CD45.2 mice and kept on antibiotic-containing water (0.2% Bacrim, Roche Germany) for the first 3 weeks. Six weeks after reconstitution these mice present a high and stable grade of blood chimerism, 96% of monocytes were of donor origin, and minimal BBB alteration^7^.

#### Fluorescent reporter mice

Thy1-CFP//LysM-EGFP//CD11c-EYFP triple heterozygous transgenic mice with multiple fluorescent cell populations have been described^21^.

Mice were housed in cages with food and water *ad libitum* in a 12 h light/dark cycle at 22 ± 1 °C. Until the end of the protocols, food was supplemented with 4% agarose jelly containing 4% glucose.

All experimental procedures were performed in accordance with the French legislation and in compliance with the European Community Council Directive of November 24, 1986 (86/609/EEC) for the care and use of laboratory animals. The research on animals was authorized by the Direction Départementale des Services Vétérinaires des Bouches-du-Rhône (license D-13-055-21) and approved by the National Committee for Ethic in Animal Experimentation (Section N°14, project APAFIS#4405-2016060811305802v2).

*EAE Induction and clinical scores (Supplemental Experimental Procedures)*

### Flow cytometry

Animals were deeply anaesthetized by i.p. injection of ketamine/xylazine (120 mg/kg; 12 mg/kg), and perfused by cardiac injection of 20 mL of cold PBS. Brains and SCs were extracted, cut into small pieces and transferred in a GentleMACS C tube with 3 mL of ice-cold dissociation buffer containing: PBS1x, 30 µg/mL DNAse I, 750 µg/mL Collagenase D (Roche Applied Science) and 425 µg/mL Collagenase V (Sigma). Mechanical and enzymatic dissociation was realized using a GentleMACS™ Octo Dissociator (Miltenyl Biotec) and a brain specific program (37C-BTDK). The suspension was then filtered (70 µM) and enzymatic reaction was blocked with EDTA (10 mM). The total cell suspension was then centrifuged on a 70%/30% Percoll density gradient. To insure a quantitative evaluation of cellular yields, CNS infiltrate Percoll gradients were simultaneously loaded with the infiltrate and a fixed number of Jurkat cells, a human T lymphocyte line identified using an anti-human antibody (hCD3). After centrifugation, myelin was removed and the immune cells containing-ring collected and washed twice. After Fc receptor blocking with 2.4G2 antibody, the cell suspension was incubated (20 min) with a mix of antibodies (see below) and dead cells labeled with SYTOX Blue (Invitrogen™) before acquisition on a 5 lasers BD LSRFortessa™. Two channels were dedicated to the detection of the fluorescent proteins LysM-EGFP and CD11c-EYFP. Data were analyzed using the BD FACSDIVA™ software. All acquisitions were done in a standardized way using application settings.

The antibodies used in the panel were: BV421 anti-CD5, BV421 anti-Ly6G, BV510 anti-IA/IE, BV610 anti-CD11b, BV650 anti CD8a, BV711 anti-CD64, PE, BV785 anti-CD11c, PE anti-CD44, PE.CF594 TCRb, PE.Cy5 anti-CD19, PE.Cy5 anti-CD161, PE-Cy5.5 anti-CD45, APC anti-human.CD3e, APC anti-F4/80, APC-Cy7 anti-Ly6C and CD45.1BV650. The antibody mix was prepared for the all experiments and the acquisition was done under strict standardized conditions.

Data are represented as mean value ± SEM of n = 3–5 individual mice per time point.

One or two-way uncorrected ANOVA were used to compare every time point of EAE animals against PBS (grey) or CFA.PTX (black) control conditions. *p < 0.05, **p < 0.01, ***p < 0.005 and ****p < 0.001.

*Blood immunophenotyping (Supplemental Experimental Procedures)*

*Glass window implantation (Supplemental Experimental Procedures)*

The window implantation protocol has previously been described in details^17–19^.

*Intravital imaging (Supplemental Experimental Procedures)*

The imaging protocol has previously been described in details^17–19^.

*Histology and immunohistochemistry (Supplemental Experimental Procedures)*

### Image Analysis

Images were analyzed using ZEN 2.1 (Zeiss), Matlab and ImageJ software. Analysis was performed on raw data, all presented images are pseudo-colored and contrast enhanced for clarity. Spectral unmixing of each channel was performed using ZEN 2.1 software (Zeiss). Home made Matlab programs were used to register image stacks to cancel out residual drifts due to animal breathing. For every mouse and every time point, cell densities were estimated manually from all the fields of view (424 × 424 microns). Densities were binned over two days (days 10 & 11, 13 & 14) and normalized to the cell densities observed on the day of EAE induction.

We developed a method to establish cell infiltration maps in the SC over development of the disease. To compare cell positions among SC slices, we designed a Matlab algorithm to normalize SC shape and cell positions relative to the center of the spinal section: each cell is located by normalized polar coordinates relative to the center-point (Supplementary Fig. 7; green dot). This method transforms each spinal slice in a standardized disk with normalized area and shape and allows establishing cell infiltration maps in the SC. An additional layer of analysis consists in subdividing the standardized spinal disk into 20 circular bands of equal areas (Supplementary Fig. 7) to precisely localize infiltrated cells and to monitor the evolution cell density evolution throughout disease progression.

All data are expressed as mean ± SEM. Statistical analysis was performed using Kruskal-Wallis and Mann-Whitney tests. p < 0.05 was used as a criterion for significance.

All data generated or analysed during this study are included in this published article (and its Supplementary Information files).

## Electronic supplementary material

Video 1

Video 2

video 3

video 4

video 5

Supplementary information
