# Supplementary material for: Diversity of innate immune cell subsets across spatial and temporal scales in an EAE mouse model
Source: Sci Rep. 2018 Mar 23;8:5146. doi: 10.1038/s41598-018-22872-y (PMC5865173; doi:10.1038/s41598-018-22872-y)
Supplement: Supplementary file 6 — Supplementary information [file 41598_2018_22872_MOESM6_ESM.pdf]

Diversity of innate immune cell subsets across spatial and temporal scales in an EAE mouse model

Céline Caravagna[1,2,7], Alexandre Jaouen[1,2,7], Sophie Desplat-Jégo[,4,5], Keith K. Fenrich[1,6], Elise Bergot[4], Hervé Luche[3], Pierre Grenot[3], Geneviève Rougon[1,2,8], Marie Malissen[3,4,8] & Franck Debarbieux*[1,2,8]

SUPPLEMENTARY FIGURES

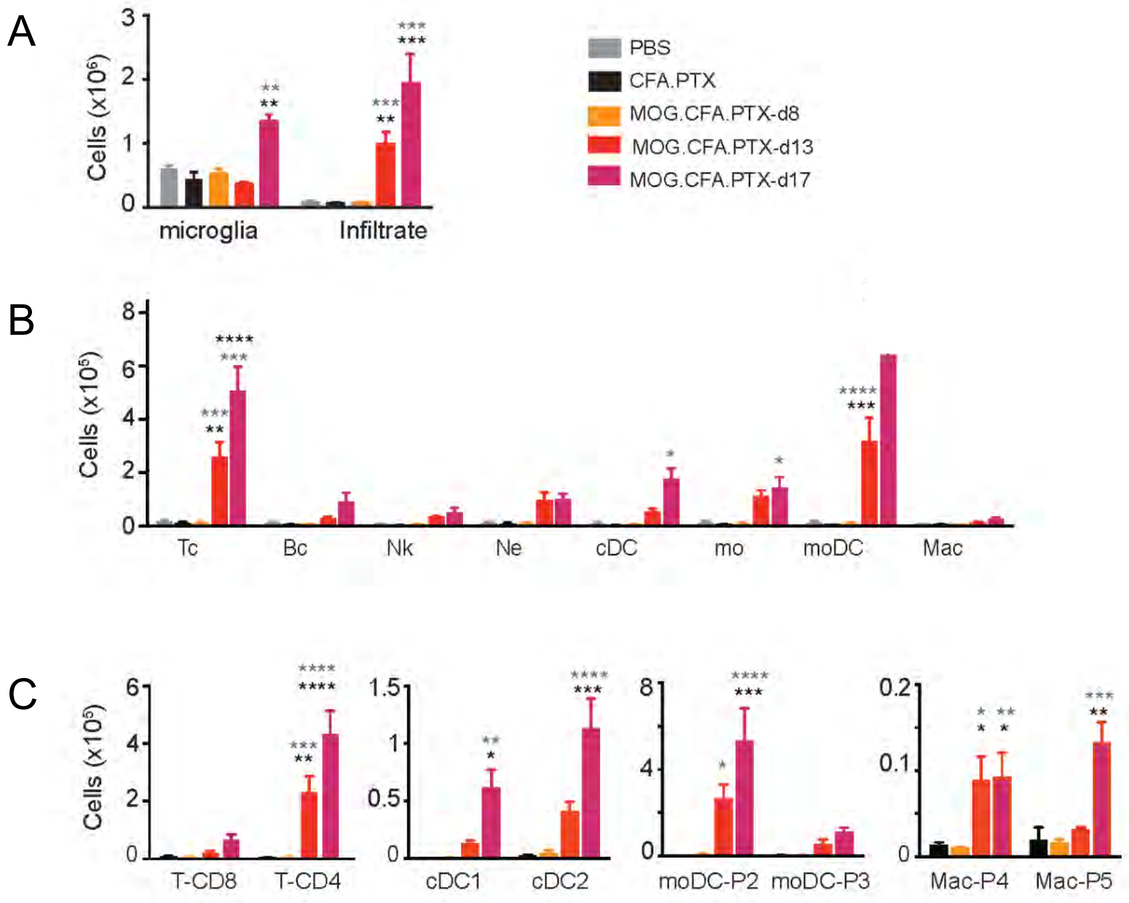

Supplementary Figure 1

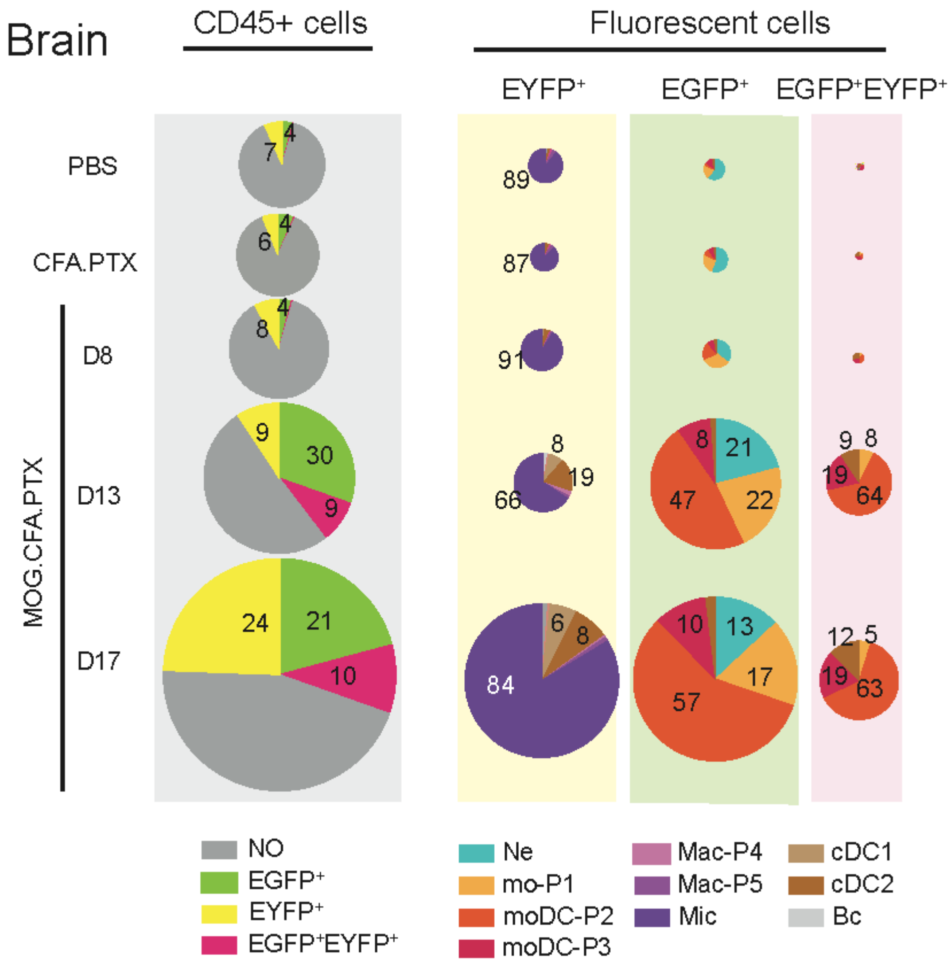

Supplementary Figure 2

A

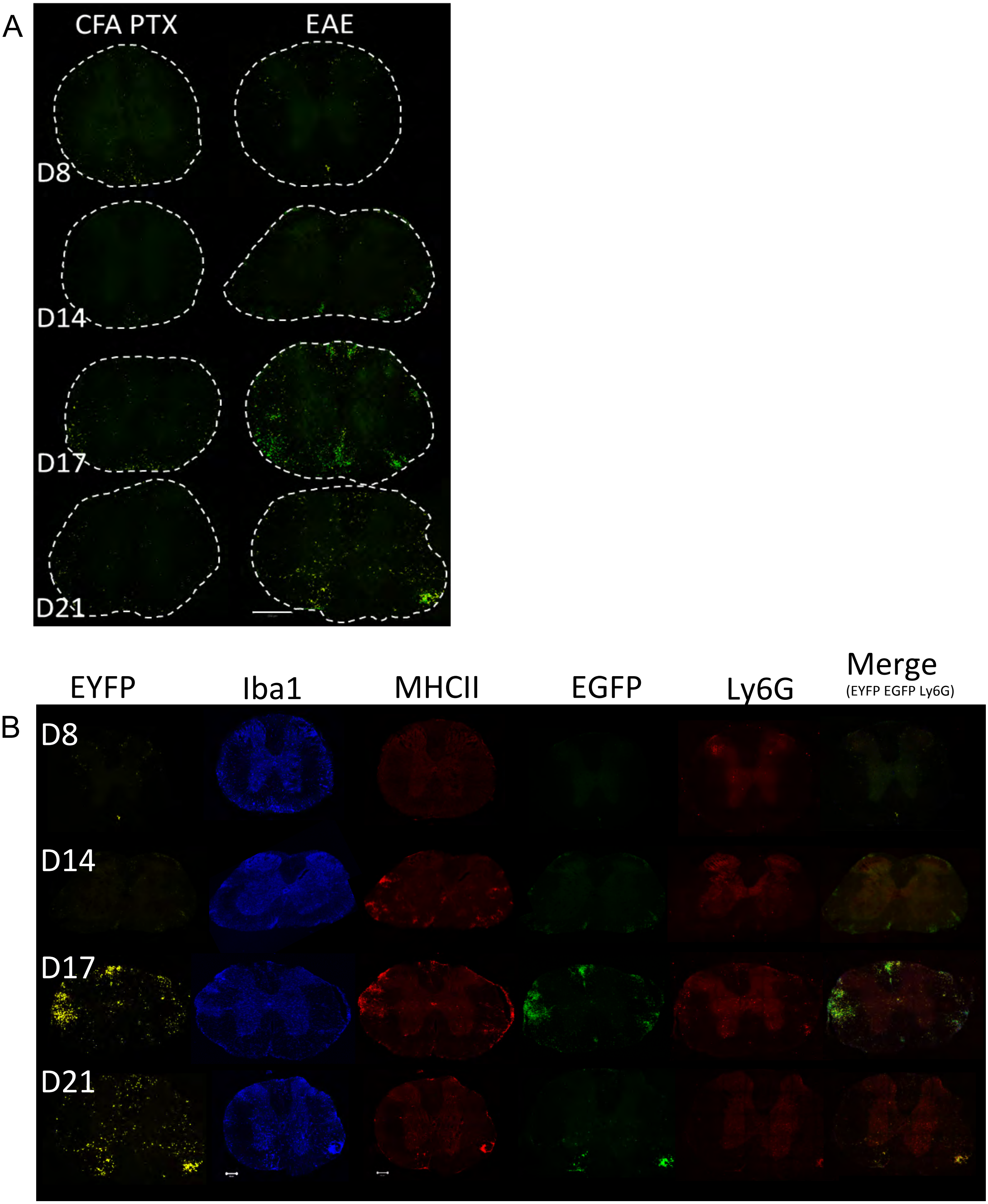

Supplementary Figure 3

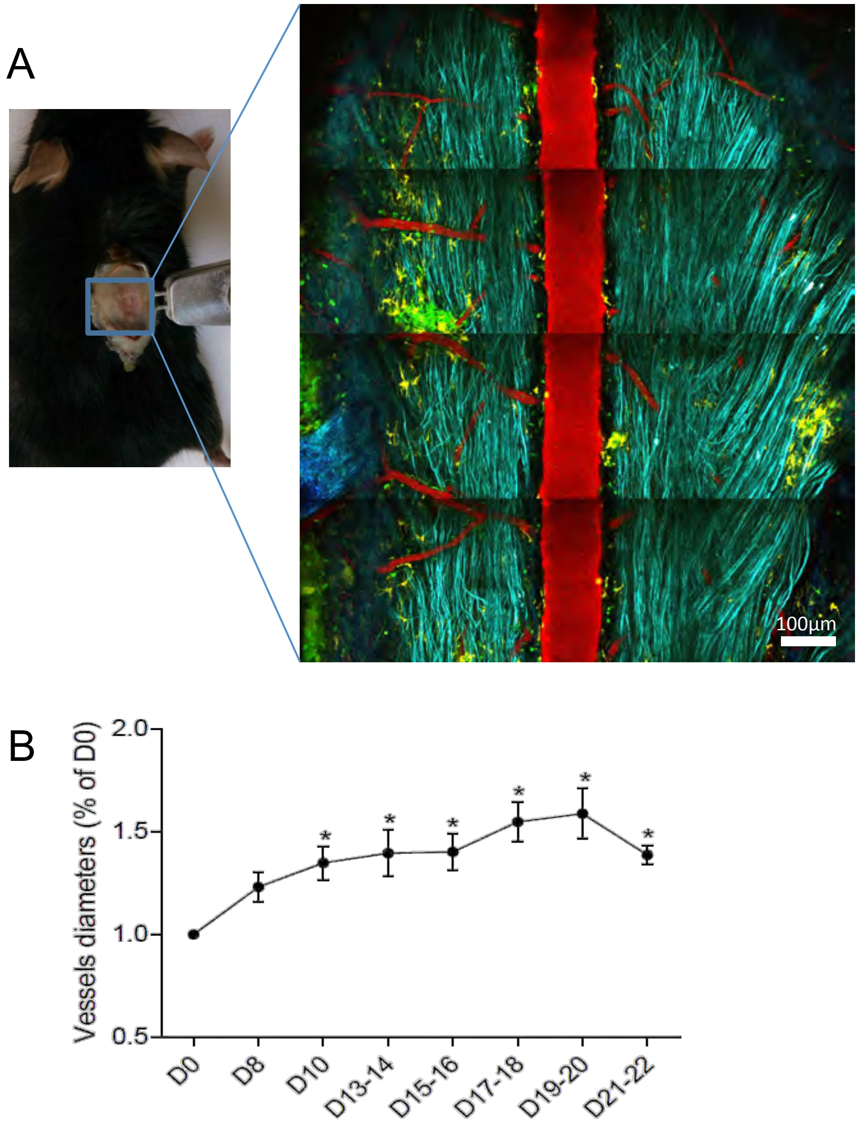

Supplementary Figure 4

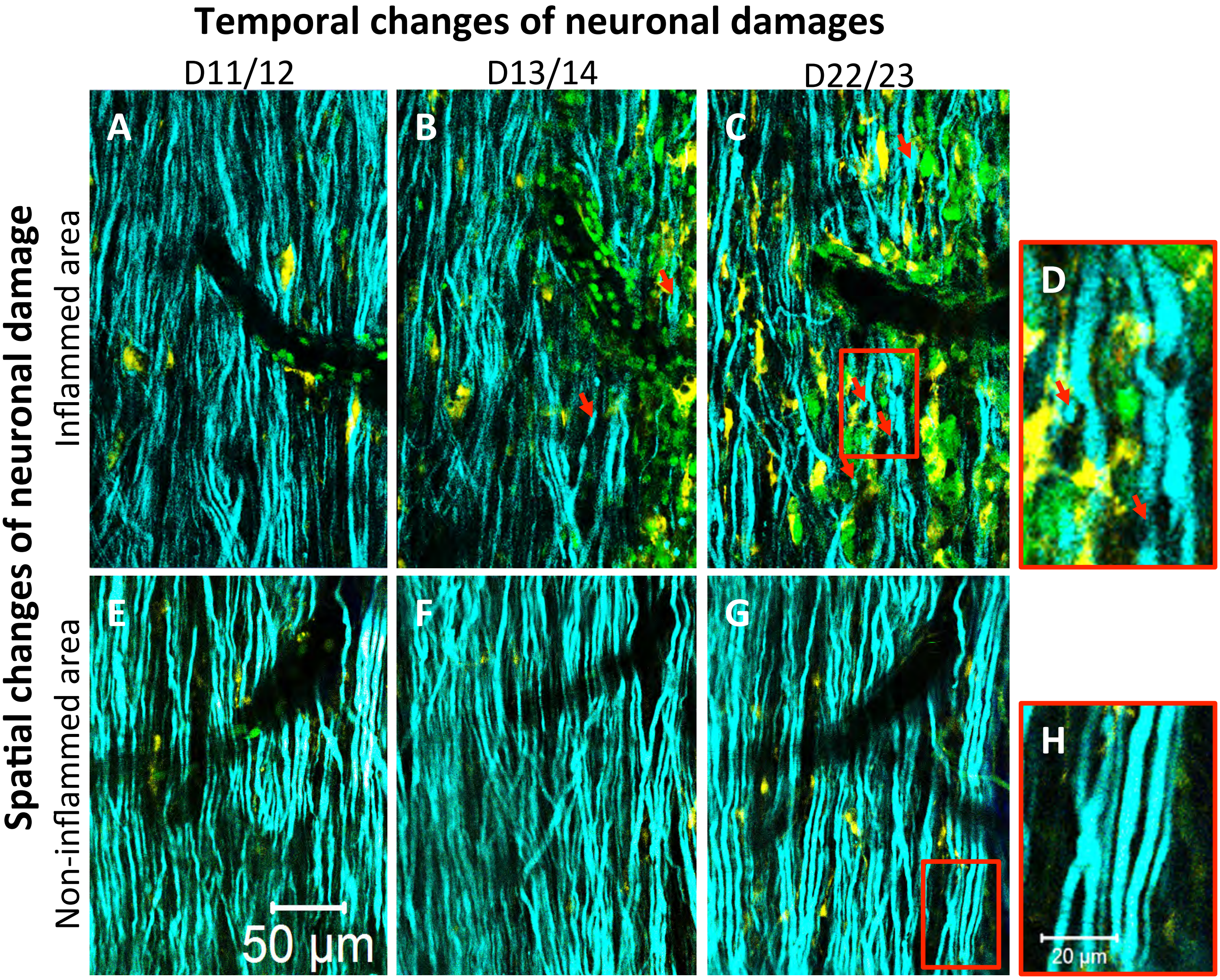

Supplementary Figure 5

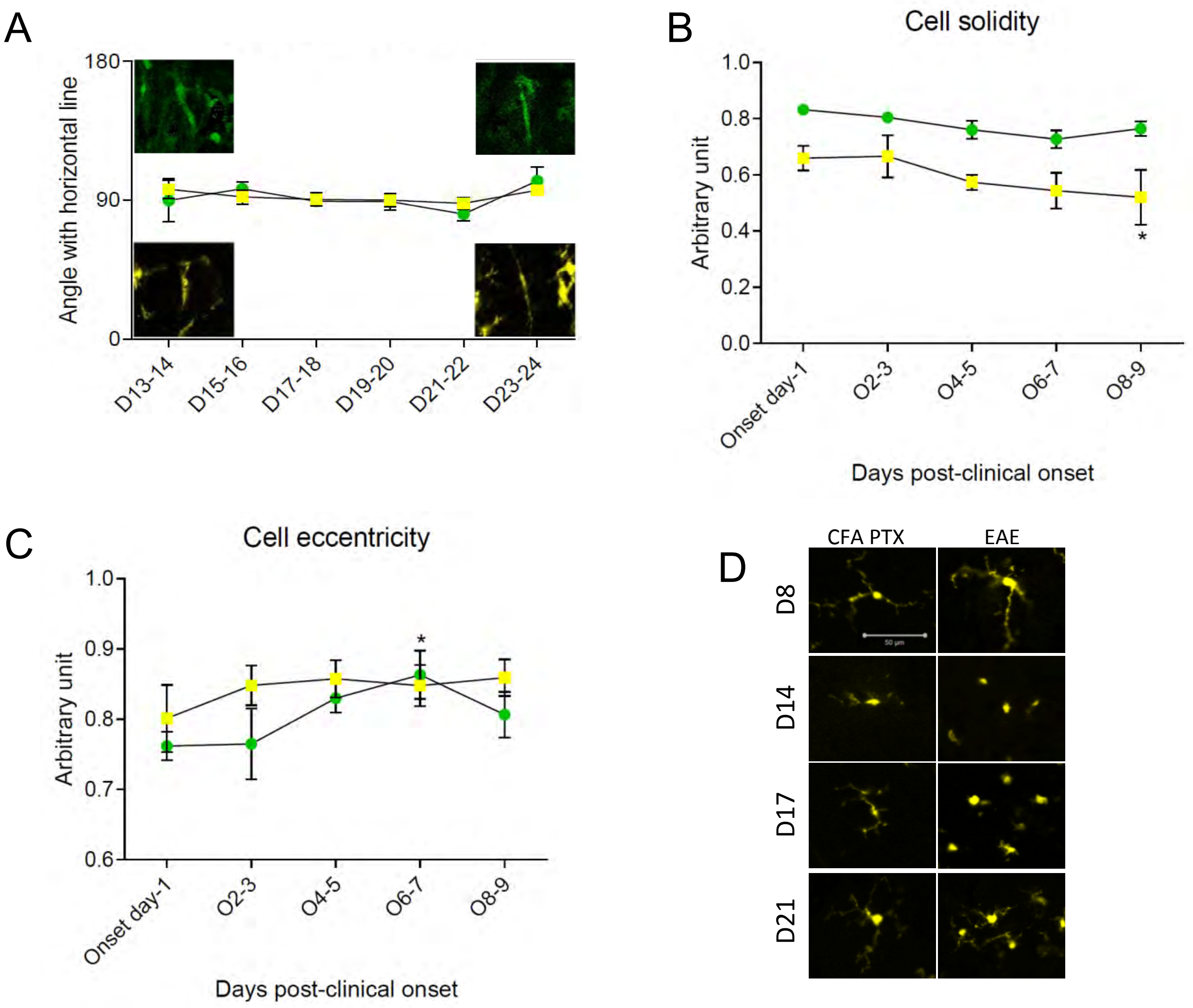

Supplementary Figure 6

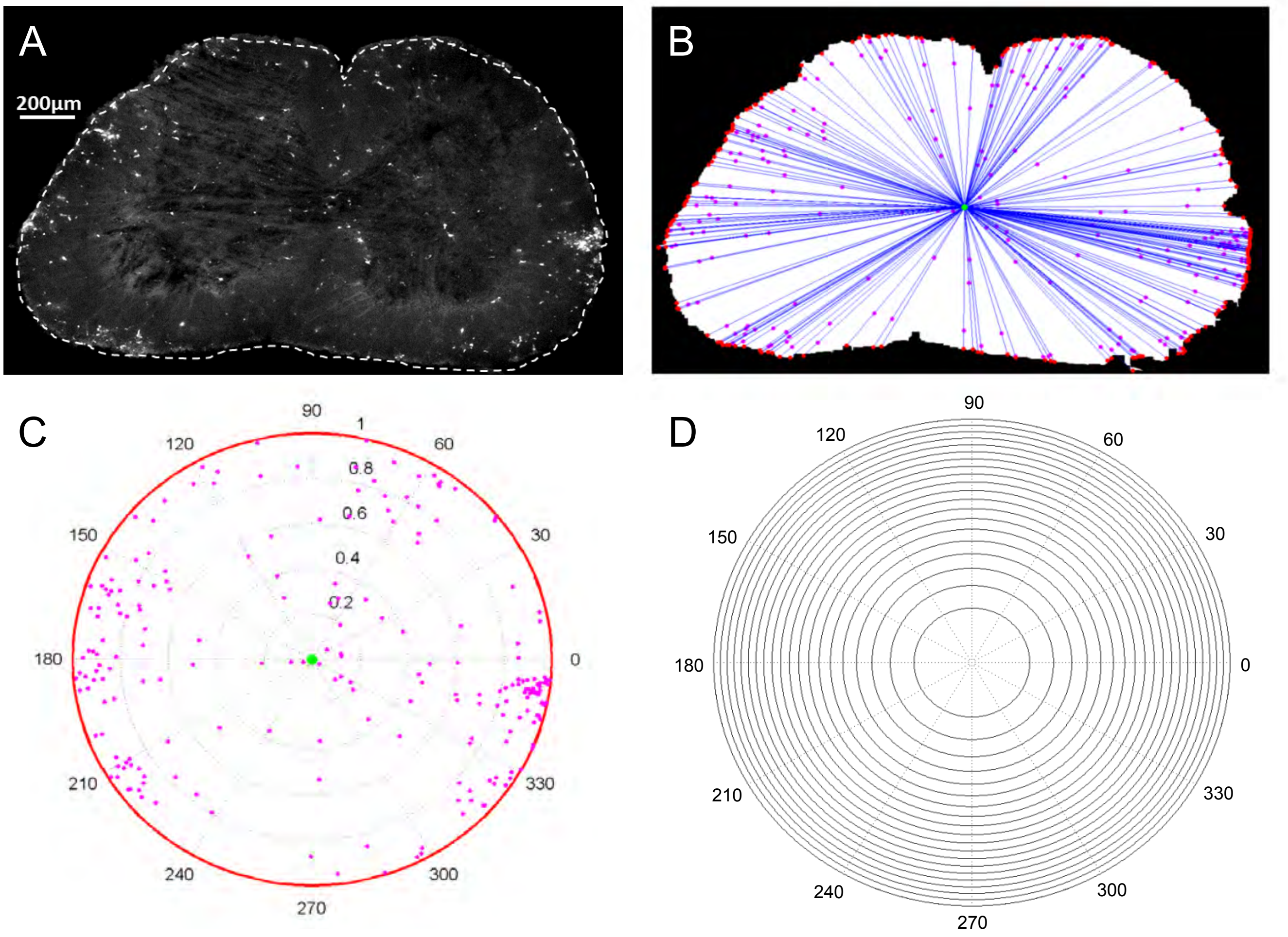

Supplementary Figure 7

Videos

V1

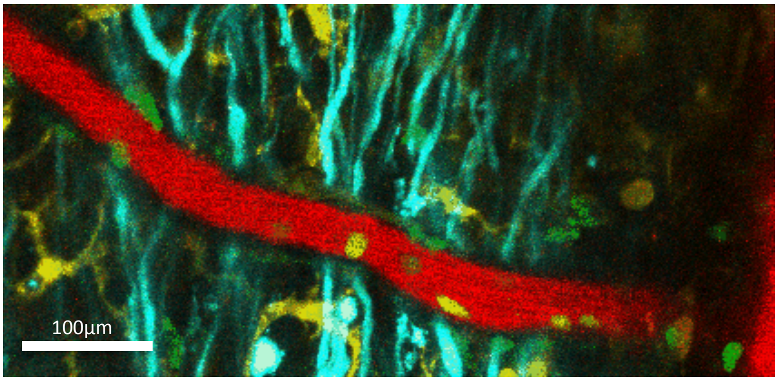

V2

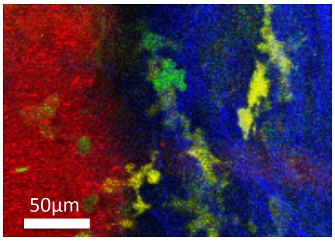

V3

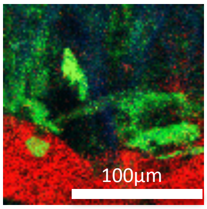

V4

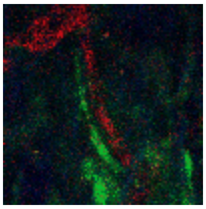

V5

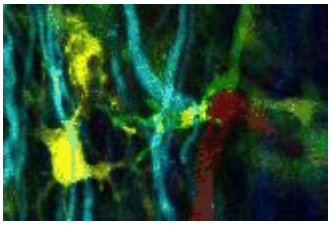

Diversity of innate immune cell subsets across spatial and temporal scales in an EAE mouse model

Céline Caravagna[1,2,7], Alexandre Jaouen[1,2,7], Sophie Desplat-Jégo[,4,5], Keith K. Fenrich[1,6], Elise Bergot[4], Hervé Luche[3], Pierre Grenot[3], Geneviève Rougon[1,2,8], Marie Malissen[3,4,8] & Franck Debarbieux*[1,2,8]

SUPPLEMENTARY LEGENDS

Legends supplementary figures

Supplementary Figure 1 : Quantification of brain infiltrated immune cells during the progression of EAE.

(A) Quantification of microglial cells versus infiltrating cells **(B)** Respective evolutions for the most representative populations of infiltrated cells. **(C)** Respective evolutions for subclasses of T cells, dendritic cells, moDCs and macrophages. n=3-5 individual mice per time point. Datas are represented as mean value ± SEM. Populations abbreviations are: T cells (Tc), B cells (Bc), NK cells (NK), Neutrophils (Ne), (conventional DCs) cDC, monocytes (mo), monocytes-derived DCs (moDC), macrophages (Mac).

Supplementary Figure 2 : Characterization of the fluorescently labeled immune cells in the brain of Thy1-CFP//LysM-EGFP//CD11c-EYFP mice.

Quantification and distribution of fluorescent cell during EAE progression. In the first column, the distribution of $CD45^{+}$ cells in $EGFP^{+}$, $EYFP^{+}$ or $EGFP^{+}/EYFP^{+}$ fluorescence is represented for control (PBS or CFA.PTX) or induced (MOG.CFA.PTX) mice at day 8, 13 or 17. The 3 others columns show for each fluorescence color, the distribution of the most represented populations. The pie chart sizes are proportional to the total number of cells. The numbers indicate the percentage of the corresponding population.

Supplementary Figure 3 : Comparison of cells recruitment in spinal slices.

(A) CFA.PTX control (left) vs EAE-induced (right) LysM-EGFP//CD11c EYFP mice. Absence of cells recruitment from day 8 to day 21 in control mice but massive recruitment in EAE induced mice. Scale bar: 500 µm.

(B) Immunohistochemistry in spinal slices from day 8 to 21 after EAE immunization. Column from left to right : $EYFP^{+}$ (yellow), $Iba1^{+}$ (blue), $MHCII^{+}$ (red), $EGFP^{+}$ (green), Ly-$6G^{+}$ (red) cells. Last column shows a merge of channels (EYFP EGFP and Ly-6G). Scale bar : 200 µm.

Supplementary Figure 4: In vivo microscopy

(A) Mice carrying a spinal glass window (left) and corresponding two-photon microscopy image (right). $EGFP^{+}$ cells (Green), $EYFP^{+}$ cells (Yellow), CFP^{+} neurons (Cyan), blood vessels (Red), type 2 collagen (Blue). (**B**) Evolution of vessel diameters as a function of post-induction time. The diameter of each vessel is the average of five neighboring measurements at each imaging session during EAE progression.

Supplementary Figure 5: Temporal and spatial axonal damage in EAE spinal cord

Evolution of morphologies and distributions of axons (cyan), activated microglial cells (yellow), monocytes and neutrophiles (green), monocyte-derived dendritic cells (yellow and green) in two different regions of the same animal : in an inflammed area (top) on D11/12 **(A)**, D13/14 **(B)** and D22/23 **(C-D)** after EAE induction; or in a neighboring non inflammed area (bottom) on D11/12 **(E)**, D13/14 **(F)** and D22/23 **(G-H). D&H** correpond to the red square region in **C&G**. Note that axons are swollen or cut (red

arrows) only when close to inflammatory plaques. Scale bar: 50μm. D, H: Scale bar: 20μm.

Supplementary Figure 6: Immune cells morphological phenotyping

(A) Privileged cell orientation of elongated cells. Horizontal is defined as 0-180° and vertical as 90-270°. Immune cells are mainly aligned vertically along axons. D = post-induction day.

(B) Solidity is a morphological parameter inversely correlated to ramification number. Cell solidity decreases from onset day until the end of experiments in EYFP$^+$ cells, and tend to decrease in EGFP$^+$ cells, indicating a tendency of cells to be more ramified days after disease onset.

(C) Eccentricity is a morphological parameter indicating the roundness of the area covered by a cell (cellular body and ramifications). EGFP$^+$ cells eccentricity increases 6-7 days after disease onset, showing the in situ maturation of these cells.

(D) Difference of EYFP$^+$ cells morphology between CFA.PTX control and EAE-induced mice. In control mice EYFP$^+$ cells remain ramified from day 8 to day 21. In EAE induced mice, morphology of EYFP$^+$ cells become ameboid at days 14 and 17 Scale bar : 50 μm.

Supplementary Figure 7: Registration of spinal cord slices

(A) Example of a spinal cord slice with visible EYFP$^+$ cells (white). Dotted line: spinal edge. Scale bar: 200μm. **(B)** Normalized coordinates of cells location. Center of the spinal cord slice (Green), position of individual cells (Magenta), spinal radius from spinal center (green dot) to spinal edge (red dot). Distance of the cell to the center is normalized by the spinal radius (blue line). **(C)** Distribution of cells (magenta) on a normalized spinal slice. Each cell has its own polar coordinates: angle (0-360°) and a distance from the center from 0 to 1. **(D)** Spinal disc is split into 20 concentric donuts of equal areas.

Supplementary video 1:

EGFP$^+$ cells migration *in vivo* over 10 min (acquired on day 17). EGFP$^+$ cells use perivascular space as a preferential migration pathway. Most of EGFP$^+$ cells inside the vessel (red) are carried away quickly by the blood flow while few of them are rolling on the endothelium. Scale bar: 100 μm

Supplementary video 2:

Migration and deformation of meningeal cells *in vivo* over 10 min (acquired on day 13). Example of a EYFP$^+$ cell and a EGFP$^+$ cell migrating in meninges (blue), by a succession of deformation. Scale bar: 50 μm

Supplementary video 3:

$EGFP^+$ cell migration in vivo over 10 min (acquired on day 16). Moving ameboid cell deformation into elongated than ameboid shapes is quite clear. Scale bar: 100 µm

Supplementary video 4:
Bipolar $EGFP^+$ cell in vivo over 10 min (acquired on day 16). Bipolar cell, here located along a blood vessel, maintains its shape and cannot be confused with moving ameboid $EGFP^+$ cell.

Supplementary video 5:
Ramified $EGFP^+$ cell in vivo over 13 min (acquired on day 21). The ramifications are still, on the contrary of ramifications of moving cells or microglial cells. These exact same cell ramifications were observed after 2h.

Diversity of innate immune cell subsets across spatial and temporal scales in an EAE mouse model

Céline Caravagna[1,2,7], Alexandre Jaouen[1,2,7], Sophie Desplat-Jégo[,4,5], Keith K. Fenrich[1,6], Elise Bergot[4], Hervé Luche[3], Pierre Grenot[3], Geneviève Rougon[1,2,8], Marie Malissen[3,4,8] & Franck Debarbieux*[1,2,8]

SUPPLEMENTARY METHODS

Supplemental Experimental Procedures

EAE Induction and clinical scores

Mice were sedated (Isoflurane 0.75-1.5% (Baxter) (v/v) in air) and subcutaneously injected with 35-55 peptide (Proteogenix) (200µg in PBS) and complete Freund's adjuvant (CFA) (Sigma) (800µg of mycobacterium tuberculosis (BD Difco)) as described (Kuerten and Angelov, 2008; Mendel et al, 1995). Induction has been completed by an intraperitoneal (i.p.) injection of pertussis toxin (PTX) (Tocris) (400ng in PBS), the same day, and two days after.
A Control group (CFA/PTX) was injected with CFA and PTX only while another (PBS) received PBS only.

Disease progression was evaluated daily by assigning a clinical score as follows: 0, no detectable signs; 0.5, flaccid tail; 1, complete tail paralysis; 2, partial hind limb paralysis; 2.5, unilateral complete hind limb paralysis; 3, complete bilateral hind limb paralysis; 3.5, complete hind limb paralysis and partial forelimb paralysis; 4, total paralysis of forelimbs and hind limbs; and 5, death. Mice typically reached a peak of disease between Days 14 and 18 after immunization followed by disease stabilization. The disease was associated with weight loss preceding the onset of clinical signs; killing of mice was required when weight loss exceeded 20 to 30% of the initial body weight or after 2 days when reaching score 4.

Histology and immunohistochemistry

Spinal cords were extracted and fixed overnight in 4% paraformaldehyde, cryoprotected in 20% sucrose. then sectioned in 25µm thick slices permeabilized in a 0.5% Triton solution. After blockage of the non-specific sites (bovine serum albumin 2%, goat serum 2%, donkey serum 2%, Triton 0.1%) slices were reacted with primary antibodies overnight at 4°C in PBS (rabbit anti-Iba1, 1/100, Wako, Cat #019-19741; rat anti-MHCII, 1/50, Ebiosciences, Cat #145321; rat anti-Ly6G, 1/100, Biolegend, Cat #127610). Secondary antibodies (goat anti-rabbit conjugated to Dylight405, 1/100, Thermo, Cat #35551; donkey anti-rat conjugated to Cy5, 1/100, Jackson Immunoresearch, Cat #712-175-150) were incubated 1h30 at RT. After washing slices were mounted with Vectashield. Imaging was performed on Carl Zeiss LSM780 confocal microscope in spectral mode, using 405, 488, 543 and 633 nm excitation wavelengths.

Glass window implantation

Briefly, mice were deeply anaesthetized with ketamine/xylazine (120 mg/kg; 12 mg/kg) (i.p.), and supplemented hourly with the same cocktail injected at a lower dose (40 mg/kg; 4 mg/kg). Following a dorsal midline incision over T12 to L2, the muscles between the spinal and transverse processes were resected using a scalpel. The animals were suspended from a spinal-fork stereotaxic apparatus (Harvard Apparatus). The dorsal musculature was further resected to expose the vertebrae, and the tips of

modified staples were inserted along the edges of the T12 and L2 and glued into place with cyanoacrylate. A modified paperclip was fixed to the staples to serve as a holding point for surgery and imaging, and a layer of dental cement was applied to form a rigid ring around the vertebrae, including staples and paperclip. Spinal processes were removed. A line of liquid Kwik-Sil (World Precision Instruments) was applied to the dura mater surface along the midline of the spinal cord, and the glass window was immediately glued and cemented over the spinal cord. Post-operative analgesia was obtained by administration of cortamethazone (0.2 mg/kg) and rimadyl (5 mg/kg) (s.c.) immediately following surgery and every two-day for 10 days. EAE was induced at least three to four weeks after glass window implantation, at a time when surgical inflammation is resumed.

Intravital imaging

Briefly, for each imaging session mice were lightly anaesthetized with 1.5% isoflurane (v/v) in air for 2 min, followed by ketamine/xylazine (100 mg/kg; 10 mg/kg). For long sessions (>1 h), animals were supplemented with 0.4–1.0% isoflurane (v/v) in air from 45 min after the start of the session until completion. To reveal blood vessels, 50µl of QDot-655 (Qtracker 655 non-targeted quantum dots; Invitrogen) (0.16pmol/µl in PBS) were injected intravenously (i.v.) at each imaging session. Throughout imaging the animals were freely breathing and the microscope chamber was warmed to 30°C. Following each imaging session the animals were returned to their cage with a piece of tissue for nesting and kept warm until they recovered from anesthesia.

A tunable femtosecond pulsed laser (Ultra II Chameleon Coherent) was coupled to a Zeiss two-photon microscope (LSM 780) equipped with a 20× water immersion objective lens (NA = 1.0) and five non-descanned detectors. The laser was tuned to 940 nm to optimize the simultaneous excitation of the fluorophore combination to be examined, and filter sets were designed to optimize the separation of the emission spectra of multiple fluorophores.

Second Harmonic signal back reflected by superficial collagen fibers was used to identify meninges. Blood vessels and axons were used as anatomical markers to find the region of interest for each animal. Tiled stack images were acquired with a field of view of 424 x 424µm with an optical sectioning of 2.5 µm over a depth typically of 80 microns below the meninges. Micropositioning allowed extraction of a conserved volume of interest throughout imaging sessions that mainly laid between 20 to 50 µm. This volume was used for quantitative analysis.

Blood immunophenotyping

Blood samples were collected into heparinized microhematocrit tubes from the retro-orbital sinuses of sedated mice. Whole blood (30 µl) was stained in Trucount™ tubes (BD Biosciences) with a combination of 11 antibodies. BD FACS™ lysing solution (BD Biosciences) was used for a 'lyse–no-wash' procedure. For each sample, a minimum of

30.10^3 $CD45^+$ cells were analyzed. The absolute number of all hematopoietic cells/microliter of blood was calculated relative to the included beads. Data were acquired and analyzed as above.

The antibodies used were: BV421 anti-CD5, BV421 anti-Ly-6G, BV510 anti-IA/IE, BV610 anti-CD11b, BV711 anti-CD19, PE anti-CCR2, PE-Cy5.5 anti CD45, PE-Cy7 anti CD43, APC anti CD161, Alexa700 anti-CD8, APC-Cy7 anti-Ly-6C.

Supplementary references:

Kuerten, S., Angelov, DN. (2008). Comparing the CNS morphology and immunobiology of different EAE models in C57BL/6 mice - a step towards understanding the complexity of multiple sclerosis. Annals of Anatomy 190, 1-15.

Mendel, I., Kerlero de Rosbo, N., Ben-Nun, A. (1995). A myelin oligodendrocyte glycoprotein peptide induces typical chronic experimental autoimmune encephalomyelitis in H-2b mice: fine specificity and T cell receptor V beta expression of encephalitogenic T cells. European Journal of Immunolgy, 25, 7.
